# Modern Analytical Techniques in Epilepsy Research

**DOI:** 10.3390/ijms27052395

**Published:** 2026-03-04

**Authors:** Katarzyna Idzikowska, Paulina Gątarek, Joanna Kałużna-Czaplińska

**Affiliations:** Institute of General and Environmental Chemistry, Faculty of Chemistry, Lodz University of Technology, 116 Zeromskiego Street, 90-924 Lodz, Poland; katarzyna.idzikowska@dokt.p.lodz.pl

**Keywords:** epilepsy, chromatography, metabolomics, biomarker, therapeutic drug monitoring

## Abstract

Epilepsy remains one of the most prevalent neurological disorders, characterised by complex aetiology encompassing genetic, structural, metabolic, and inflammatory factors. Despite advances in neuroimaging and neurophysiological diagnostics, there is a persistent lack of sensitive and specific biomarkers to enable early diagnosis, risk stratification, and monitoring of therapeutic efficacy. Key epilepsy biomarkers include neurotransmitters, energy–related compounds, tryptophan pathway metabolites, and choline derivatives. Their determination employs liquid chromatography coupled with tandem mass spectrometry (LC–MS/MS), high–performance liquid chromatography (HPLC) with electrochemical or fluorescence detection, gas chromatography with tandem mass spectrometry (GC–MS/MS), high–resolution mass spectrometry (HRMS), and proton nuclear magnetic resonance (^1^H–NMR) spectroscopy, revealing metabolic disturbances in neurotransmission, energy metabolism, and oxidative stress associated with epileptogenesis. Among these techniques, LC–MS/MS currently provides the highest analytical sensitivity and specificity for quantifying low–abundance epilepsy–related metabolites, while HPLC with conventional detection remains a simpler and more cost–effective alternative for routine clinical laboratories. This review presents the current state of knowledge regarding chromatographic techniques applied to the analysis of mentioned metabolites, as well as therapeutic drug monitoring of antiepileptic drugs. Key sample preparation stages are also discussed. Various biological matrices–plasma, serum, urine, cerebrospinal fluid (CSF), dried blood spots (DBSs), and brain tissue—are evaluated. Novel approaches are also presented, including hair samples, microsampling techniques, and headspace analysis of volatile metabolites. Chromatographic techniques constitute the foundation of contemporary metabolomic research in epileptology, enabling biomarker identification and supporting personalised medicine. Further standardisation and translational validation remain necessary, as current evidence is insufficient for routine clinical implementation.

## 1. Introduction

Epilepsy is a chronic neurological disorder characterised by recurrent, spontaneous epileptic seizures resulting from abnormal electrical activity in the brain. According to the World Health Organization (WHO), epilepsy is one of the most common neurological diseases, affecting over 50 million people worldwide [1], with approximately 2.4 million new cases recorded annually. Although the WHO does not specify the precise percentage of children among those affected by epilepsy, available evidence indicates that epilepsy is one of the most common neurological disorders in the paediatric population. In developed countries, the annual incidence of epilepsy is approximately 50 per 100,000 persons, whilst in developing countries this rate may be up to twice as high [2]. Recent research on the aetiology of epilepsy has focused on genetic, neurological, inflammatory, and metabolic factors [3]. Genetic factors are particularly important, as many genes associated with idiopathic forms of epilepsy encode ion channels crucial for neuronal function. Mutations in these genes may lead to abnormal synchronisation and excessive excitability of neuronal networks [4]. Structural factors, including traumatic brain injuries and vascular diseases such as stroke are also important causes. These alterations may lead to structural and functional modifications in the brain that increase susceptibility to epileptic seizures. Inflammatory processes, both infectious and non–infectious, may contribute to epileptogenesis by inducing inflammatory reactions in the brain. Infections of the central nervous system (CNS) represent a significant risk factor for epilepsy development [5,6], as neural tissue damaged by dysregulated inflammation poses a high risk for neurological disease [7]. Metabolic disturbances also play a significant role in epilepsy pathogenesis. Metabolic encephalopathy—a serious disorder of brain function resulting from metabolic abnormalities rather than primary structural damage—may lead to epilepsy development [8]. Despite being highly developed, current diagnostic pathways remain insufficient and present several challenges. Accurate seizure documentation is crucial for medical assessment; however, patients frequently fail to document all seizures, impeding accurate assessment of their frequency and character [9]. The diverse symptom presentation of epilepsy often leads to diagnostic errors, significantly impacting treatment, particularly in challenging cases such as insular epilepsy [10]. Furthermore, continuously advancing genomic technologies reveal the genetic complexity of epilepsy, necessitating novel approaches to classification and, consequently, to therapy [11]. Given these limitations, there is a clear need to identify biomarkers that could assist in more accurate diagnosis, prognosis, and disease monitoring [12]. Biomarkers play a crucial role in both diagnosis and treatment optimisation, enabling better understanding of the disease and adaptation of therapy to individual patient needs. Therapeutic drug monitoring, in particular, is essential for therapy optimisation and minimisation of adverse effects.

The development of novel diagnostic methods is, therefore, imperative to improve patients’ quality of life and enable individualised therapeutic selection. In epileptology, this represents an area of continuous development aimed at identifying characteristic biomarkers and establishing precise validated methods for their determination. In response to growing diagnostic and therapeutic challenges, there is an urgent need to develop and validate innovative diagnostic strategies that could eventually be implemented in routine laboratory practice. An optimal test should utilise readily accessible biological material whilst maintaining high diagnostic specificity and sensitivity. Such a biomarker would enable risk stratification for epilepsy development, early and precise differential diagnosis of individual epileptic disorder types, and prediction of treatment response. This individualised diagnostic approach would represent significant progress towards personalised medicine in epileptology, contributing to therapeutic process optimisation and improvement of patients’ quality of life [13,14,15]. The aim of this review is to provide a systematic and critical discussion of current achievements in the application of chromatographic techniques in epilepsy research, with particular emphasis on their role in biomarker identification, characterisation of metabolomic disturbances, and therapeutic monitoring of antiepileptic drugs. The review encompasses both classical methods, including liquid chromatography coupled with mass spectrometry (LC–MS), high–performance liquid chromatography (HPLC), and gas chromatography coupled with mass spectrometry (GC–MS), and advanced analytical techniques, such as liquid chromatography coupled with tandem mass spectrometry (LC–MS/MS), chromatography coupled with high–resolution mass spectrometry (HRMS), microsampling techniques, dried blood spot (DBS) analyses, and targeted and untargeted metabolomic approaches.

This work aims to evaluate the utility of these methods in epilepsy pathogenesis research, including the analysis of neurotransmitters, energy metabolites, gut–brain axis compounds, and the tryptophan–kynurenine pathway, as well as their application in differentiating clinical phenotypes and identifying novel candidates for diagnostic and prognostic biomarkers. An additional objective is to assess the translational potential of the discussed chromatographic techniques, encompassing their possible implementation into routine laboratory diagnostics, early risk stratification, therapy personalisation, and monitoring of treatment efficacy and safety. Finally, this review identifies key areas requiring further validation and standardisation that will determine the future application of advanced chromatographic techniques as tools supporting personalised medicine in epileptology.

## 2. Preparation of Biological Samples

Body fluids are an invaluable medium in research on neurological diseases, providing diagnostic and prognostic information. Their analysis may yield novel and more specialised biomarkers that assist in the diagnosis and prognosis of epilepsy, which is particularly important given the heterogeneous nature of this disease [16]. Studies of fluids such as blood, serum, plasma, and cerebrospinal fluid (CSF) provide valuable information regarding the aetiology, diagnosis, and prognosis of applied therapies. Studies involving blood, serum, and plasma offer the advantage of less invasive sample collection compared to CSF and permit ready analysis at various time points [17].

However, metabolomic research in epilepsy faces certain challenges, particularly when utilising cerebrospinal fluid. CSF collection is invasive and carries considerably greater risk of complications than venous blood collection. Nevertheless, it may provide valuable information regarding pathological metabolic processes that cannot be obtained from peripheral samples.

Due to the complexity of biological matrices, effective sample preparation is crucial not only for minimising interference and eliminating interfering substances but also for preserving metabolite integrity and ensuring representativeness of the analysed metabolic profile. This process encompasses a series of stages—from proper sample collection and storage, through selection of an appropriate extraction method, to purification and stabilisation—each significantly impacting the final quality of analytical data. Inappropriate sample preparation may lead to the degradation of key compounds, appearance of analytical artefacts, and reduction in method sensitivity and selectivity, consequently limiting the reliability and reproducibility of metabolomic analysis results. The individual stages of sample preparation for metabolomic analysis are described below. Deproteinisation of biological samples prior to analysis is a crucial stage of sample preparation for several important reasons. Primarily, it prevents damage to analytical equipment, as proteins may deposit on chromatographic columns, capillaries, or detectors, shortening their lifespan and reducing efficiency. This process also eliminates interference, since proteins may disrupt analyte detection at low concentrations by masking signals or generating false positives. Protein removal simplifies the sample matrix, thereby improving the selectivity and sensitivity of target analyte determination.

Enhancement of sample stability represents another significant aspect, as certain proteins, such as enzymes, may cause analyte degradation during storage. This procedure also improves the reproducibility of both results and the entire analytical procedure. Following protein removal, concentration of analytes present at low levels becomes more feasible, which is crucial for metabolomic analyses.

Deproteinisation techniques include precipitation using organic solvents (such as acetonitrile or methanol), acids (e.g., sulphosalicylic acid, formic acid, or trifluoroacetic acid), and ultrafiltration methods [18]. Analyte properties represent a key factor: physicochemical characteristics including polarity, molecular mass, stability, and solubility, determine the choice of solvents and methods. For lipophilic compounds, liquid–liquid extraction with non–polar solvents is frequently employed, whilst for polar compounds, solid–phase extraction (SPE) with appropriately selected sorbents proves more effective, simultaneously enabling analyte concentration in the sample. Among the extraction methods for epilepsy–associated metabolites listed in Table 1, liquid–liquid extraction with methanol or dichloromethane was most frequently employed, occasionally assisted by ultrasonication. An interesting technique is air–assisted liquid–liquid microextraction (AALLME), which involves repeated withdrawal and introduction of air into a mixture of sample and extraction solvent using a syringe. This causes intensive dispersion of the solvent in the form of fine droplets, increasing the contact surface area between phases and extraction efficiency whilst using minimal solvent volumes [18].

**Figure 1:**
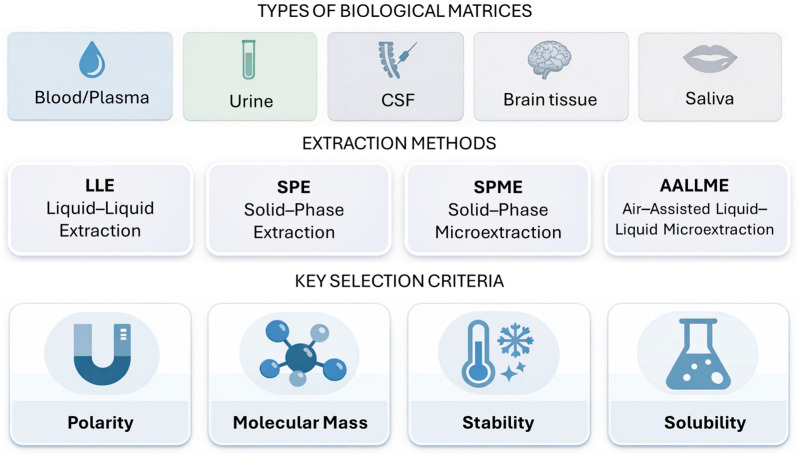
Overview of biological matrices, extraction techniques, and key physicochemical parameters guiding modern analytical strategies in epilepsy research/the search for epilepsy biomarkers.

The matrix type is also significant: blood, urine, saliva, cerebrospinal fluid, and brain tissue differ in their contents of proteins, lipids, and salts, which influences technique selection. Samples with high protein content (serum, plasma) frequently require preliminary deproteinisation, whilst urine may require enzymatic hydrolysis. Matrix complexity determines method selectivity: fluids containing numerous endogenous compounds require more selective extraction methods, such as SPE with mixed–mode phases, solid–phase microextraction (SPME), or biocompatible solid–phase microextraction (BioSPME) [19]. In certain cases, brain tissues undergo only homogenisation with the addition of methanol or perchloric acid, followed by centrifugation and immediate analysis [20,21]. A summary of the extraction methods mentioned above in the analysed metrics and process condition selection indicators is presented in Figure 1.

Derivatisation in chromatographic analysis is a process of chemical modification of analyte molecules to impart properties more suitable for instrumental analysis. The choice of derivatisation method depends primarily on the chemical structure of the analysed compounds and the chromatographic technique employed [22].

In gas chromatography (GC), derivatisation is used mainly to increase the volatility of non–volatile or poorly volatile compounds, improve thermal stability, reduce polarity by blocking functional groups (such as –OH, –COOH, –NH_2_, and –SH), and enhance chromatographic resolution.

In liquid chromatography (LC), derivatisation serves primarily to increase detection sensitivity by introducing chromophoric groups for ultraviolet–visible (UV–VIS) detection or fluorophoric groups for fluorescence detection, as well as to improve separation of structurally similar compounds. The most frequently employed derivatisation methods include the following:

- Silylation together with methoximation (methoxyamines in pyridine, trimethylchlorosilane (TMCS), and n–methyl–n–(tert–butyldimethylsilyl)trifluoroacetamide (MTBSTFA));
- Hydrochloric acid (HCl) in n–butanol, which catalyses the esterification reaction of carboxyl groups in amino acids and organic acids, forming volatile butyl esters with improved chromatographic properties, enabling their effective analysis primarily by GC;
- OPA/βME, a derivatisation system utilising o–phthalaldehyde (OPA) in combination with 2–mercaptoethanol (βME) to convert primary amino groups into highly fluorescent isoindole derivatives, enabling sensitive detection of compounds containing the –NH_2_ group in LC. Similar activity is exhibited by the reagents: PNP (paranitrophenyl) and FMN (fluoromethylnitrophenylpropionyl), as well as incubation with citrate–phosphate buffer and 2–chloroacetaldehyde;
- Semicarbazide and glycine as derivatisation reagents for converting carbonyl compounds (aldehydes and ketones) into corresponding semicarbazones and hydrazones, increasing their thermal stability, inter alia, during GC analysis [22,23,24,25,26].

The final frequently mentioned stage of sample preparation is reconstitution in solvent. This stage of analytical sample preparation involves dissolving the dry residue obtained after evaporation of the previous solvent. This process enables a change in the environment in which the analytes are present—from one that was optimal for extraction to one that is compatible with the chromatographic system (e.g., from an organic solvent to a mobile phase). Reconstitution also allows for the concentration of analytes by dissolving the dry residue in a smaller volume of solvent than the initial sample. Additionally, it facilitates the elimination of interferents that may not dissolve in the newly selected solvent and enables adjustment of the pH and other sample parameters to match the conditions of instrumental analysis, thereby improving chromatographic peak shape and detection sensitivity. Among the most frequently used reconstitution solvents are methanol (either pure or diluted with water), formic acid, and phosphate buffer [27,28,29]. Table 1 presents a detailed characterisation of sample preparation methods employed in the research on the identification of diagnostic biomarkers of epilepsy, with consideration of the type of tissue analysed and the chromatographic technique applied.

**Table 1:** Detailed characterisation of sample preparation in the search of diagnostic markers with consideration of physiological fluid and/or tissue and the chromatographic technique applied.

No.	Human/ Animal Model	Tissue	Technique	Preparation	Determined Analytes	Ref.
1	A	Brain	HPLC	Purification: sonication, centrifugation 40 min, filtration (PTFE filter with pore diameter of 0.22 µm), IS–DHBA; homogenisation in neurotransmitter–stabilising solution of 0.1 M perchloric acid and 0.1 mM sodium metabisulphite	Dopamine, noradrenaline, serotonin, 3,4–dihydroxyphenylacetic acid, homovanillic acid, 5–hydroxyindoleacetic acid, vanillylmandelic acid, 3–methoxytyramine, 4–hydroxy-3–methoxyphenylglycol	[30]
2	H	Brain (post–mortem)	LC	Extraction: ethanol with phosphate buffer, sonication, centrifugation	Metabolites of choline metabolism	[31]
3	H	Plasma/urine	HPLC	Deproteinisation: 6.3% sulphosalicylic acid and ACN, derivatisation: 3N HCl in n–butanol at 65 °C for 30 min, drying, reconstitution: water/methanol (70:30) IS: d9–pipecolic acid	α–Aminoadipic semialdehyde, piperideine–6–carboxylate, pipecolic acid	[32]
4	H	Serum	GC	Extraction: methanol:H_2_O, 8:1, *v*/*v*, centrifugation, derivatisation: methoximation: 30 µL methoxyamine HCl in pyridine, incubation 16h at room temp., silylation: 30 µL MTBSTFA + 1% TMCS, incubation 1 h at 37 °C	Amino acids: glutamate, proline, asparagine, cysteine; fatty acids: palmitic acid, linoleic acid, stearic acid, elaidic acid	[33]
5	A	Plasma, hippocampus	HPLC	Homogenisation: sonication in 0.1% NH_4_OAc, modified liquid–liquid extraction (Matyash method)	Nucleosides, nucleotides, purine and pyrimidine metabolites, organic acids and their derivatives, peptides and amino–acid–related metabolites, ceramides, glucosylceramides, diacylglycerols, phosphatidylcholines, triacylglycerols and vitamin D metabolites and its derivatives	[34]
6	H	CSF	UPLC	Deproteinisation: ACN/methanol (9:1, *v*/*v*) + 0.1% formic acid, incubation in darkness 20 min, centrifugation, evaporation under nitrogen 60 °C, reconstitution: 0.1% formic acid	Pyridoxal–5′–phosphate, pyridoxal, pyridoxine, pyridoxamine, pyridoxic acid	[35]
7	H	Serum	GC	Extraction: methanol:H_2_O (8:1, *v*/*v*), centrifugation, evaporation under nitrogen 70 °C, derivatisation: methoximation 16 h room temp, trimethylsilylation 37 °C 1 h	GABA, glutamate, dopamine, serotonin	[21]
8	H	Microdialysate	HPLC	OPA derivatisation prior to HPLC analysis	GABA, glutamate, glutamine	[28]
9	A	Plasma	LC	Mixed with methanol in ratio 1:5 (*v*/*v*), internal standard: gabapentin, centrifugation	Homostachydrine	[20]
10	A	Hippocampus, frontal cortex	LC	Homogenisation in methanol, centrifugation	Homostachydrine	[20]
11	A	Brain	HPLC	Homogenisation in 10% perchloric acid, centrifugation	GABA, glutamate, dopamine, serotonin	[21]
12	H	Cerebrospinal fluid, plasma	UPLC	Centrifugation, liquid–liquid extraction with methanol, drying, dissolution in mobile phase (H_2_O/ACN 50:50), filtration, SPE	33 Dipeptides and amino acids	[36]
13	A	Hippocampus–microdialysate	HPLC	Derivatisation: incubation of sample with citrate–phosphate buffer and 2–chloroacetaldehyde, 40 min, 80 °C	ATP, ADP, AMP, adenosine	[37]
14	A	Plasma	LC	Deproteinisation: acetonitrile, mixing, centrifugation, supernatant diluted with solution of water and 0.1% formic acid	Aminoadipate; saccharopine; pipecolate; glutamic acid; piperideine–6–carboxyl acid; pyridoxal–5–phosphate	[38]
15	A	Urine	GC	SPME extraction	VOCs	[39]
16	H	Plasma, urine, dried blood spot	LC	Plasma: deproteinisation: ACN containing IS (d_3_–AAA, d_9_–PA), mixing, centrifugation, evaporation with nitrogen, dissolution (H_2_O:methanol, 70:30)	α–Aminoadipic semialdehyde, α–aminoadipic acid, pipecolic acid	[40]
17	H	Serum	UHPLC	Deproteinisation: methanol with chloromethylalanine, ultrasonic extraction, incubation, centrifugation	Malathion monocarboxylic acid, serylproline, asparagylthreonine, 7–methyl–3–oxo–6–octenoyl–coenzyme A, aspartyl-phenylalanine, phenylalanylphenylalanine, artonol B	[41]
18	A	Hippocampus	UPLC	Homogenisation in methanol with H_2_O (1:1, *v*/*v*), centrifugation, drying in CentriVap^®^ (40 °C, 6 h), MTBE/methanol extraction (3:1, *v*/*v*)	Polar metabolites: glutamine, N–acetyl–L–aspartate, tryptophan, adenosine, glucose–6–phosphate, fructose–6–phosphate, fructose–1,6–bisphosphate, dihydroxyacetone phosphate, 2–phosphoglycerate, 3–phosphoglycerate, phosphoenolpyruvate, pyruvate, citrate, 2–oxoglutarate, succinate, fumarate, malate; non–polar metabolites: cholesterol, triglycerides, phosphatidylcholine, phosphatidylserine, free fatty acids, cholic acid, prostaglandins, leukotrienes, anandamide, 2–arachidonoylglycerol	[42]
19	A	Somatosensory cortex–microdialysate	UHPLC	Derivatisation: OPA/βME prior to injection, internal standard: glutamate, GABA	GABA, glutamate	[43]
20	H	Dried blood spot	LC	Samples dried on Whatman 903 cards, 3 mm disc rehydrated in tris–phosphate buffer, sonication, incubation with PNP and FMN 30 min 37 °C, reaction stopped: trichloroacetic acid, centrifugation	Pyridoxal–5–phosphate, pyridoxine–5–phosphate, pyridoxamine–5–phosphate	[44]
21	A	Hippocampus (surgically excised in vivo)	HPLC/LC	Homogenisation in perchloric acid, centrifugation	Tryptophan, kynurenine, kynurenic acid, PLP, quinolinic acid, glutamate, GABA	[45]
22	H	Cerebrospinal fluid	HPLC	Derivatisation: semicarbazide/glycine, incubation 40 °C 30 min, deproteinisation with perchloric acid, filtration	Pyridoxal 5′–phosphate, pyridoxal, 4–pyridoxic acid	[46]
23	A	Striatum, substantia nigra	UPLC	Glycoprotein isolation (homogenisation in Tris–SDS buffer, centrifugation, supernatant purification) N–glycan isolation (enzymatic digestion with PNGase F, glycan lyophilisation, incubation at 65 °C for 1.5 h, removal of excess reagents and dissolution in water)	N–glycans	[47]
24	A	Extracortical fluid–microdialysate	GC	Separation on ion–exchange column (elution with water/hydrochloric acid/NH_4_OH), freezing, lyophilisation, derivatisation: (MTBSTFA)	Glutamate, glutamine	[48]

A—animal; ACN—acetonitrile; ADP—adenosine diphosphate; AMP—adenosine monophosphate; ATP—adenosine triphosphate; d_3_–AAA—trideuterio–2–aminohexanedioic acid; d_9_–PA—nonadeuteriohexadecanoic acid; DHBA—3,4–dihydroxybenzylamine; GABA—γ–aminobutyric acid; H—human; H_2_O—water; IS—internal standard; MTBE—methyl tert–butyl ether; NH_4_OAc—ammonium acetate; NH_4_OH—ammonium hydroxide; PLP—pyridoxal 5′–phosphate; PNGase F—peptide–n^4^–n–acetyl–β–d–glucosaminyl)asparagine amidase; PTFE—polytetrafluoroethylene; Tris–SDS buffer—tris(hydroxymethyl)aminomethane–sodium dodecyl sulfate buffer; UHPLC—ultra–high–performance liquid chromatography; UPLC—ultra–performance liquid chromatography; VOCs—volatile organic compounds.

Analysis of body fluids allows for monitoring of treatment efficacy, including pharmacological treatment methods. However, it is worth drawing attention to other readily accessible tissues and body fluids that would enable rapid, straightforward control of drug concentration levels, such as the previously mentioned saliva and DBS [49,50,51]. An interesting approach to long–term monitoring of drug concentration levels is the use of hair as a biological matrix, which allows for the assessment of chronic exposure and treatment adherence. In a study conducted by Karaś-Ruszczyk et al. [52], LC–MS/MS was employed to determine levetiracetam (LEV) concentrations in the hair of patients with epilepsy. The study group comprised 47 patients, including 27 women and 20 men, with a mean age of 37.4 ± 11.9 years. Samples were collected from the occipital region (1 cm from the scalp) and subsequently subjected to solid–liquid extraction (SLE) and analysis. The extraction involved incubation of 20 mg of cut hair in 1 mL of methanol at 60 °C for 1 h, after which the supernatant was collected and deproteinised by addition of acetonitrile with an internal standard. Following centrifugation and reconstitution with the mobile phase, the sample was prepared for LC–MS/MS analysis. The method for determination of LEV in the hair of patients with epilepsy was validated, being characterised by the following validation parameters: linearity range of 0.05–5 µg/mg, lower limit of quantification (LLOQ) of 0.05 µg/mg, and recovery of 98.3–99.8%. One of the advantages of hair analysis is the possibility of eliminating result manipulation through, for example, taking medication only before a follow–up visit. Authors utilising LC–MS/MS demonstrated a very strong correlation of levetiracetam concentrations between saliva and plasma, whereas the relationship between hair and plasma was weak and showed no association with drug dose. Methodological limitations include the absence of full bioanalytical validation, lack of longitudinal studies, and lack of preanalytical standardisation, which significantly restricts the clinical interpretation of results obtained from hair. It should also be noted that the research group was too small to draw global conclusions for use in diagnostic tests. Consequently, hair analysis may have only limited exploratory value in assessing chronic exposure, but at the current stage does not constitute a reliable tool for therapeutic drug monitoring or treatment outcome assessment [52]. A novel approach in drug administration was presented through the application of an automated system developed in an animal model, utilising HPLC with ultraviolet (UV) detection for carbamazepine (CBZ) and its active metabolite—carbamazepine–10,11–epoxide (CBZ–E)—in rat plasma. This study was conducted on 13 Sprague–Dawley rats (190–280 g), demonstrating that four–times–a–day dosing of CBZ effectively maintains therapeutic concentrations and that CBZ–E accumulates more strongly than previously reported. The method was validated, being characterised by the following parameters: precision (coefficient of variation (CV) intra–day 2.3–7.5%, CV inter–day 3.2–8.1%), linearity (R^2^ ≥ 0.998), and recovery of CBZ (79.9–87.9%) and CBZ–E (92.8–94.5%), confirming its utility in pharmacokinetic analysis. The application of an automated drug delivery system increases result reproducibility by eliminating manual errors, which makes this model a promising tool for research on the pharmacokinetics of other antiepileptic drugs. Despite acceptable validation parameters, methodological limitations (UV detection, small sample size, and animal model) and the absence of control of correlation with antiepileptic effects significantly restrict the translational value of the results. Consequently, the proposed system should be regarded as a methodological tool for standardising pharmacokinetic studies rather than as a solution with direct clinical or diagnostic potential [53]. Table 2 presents a detailed compilation of sample preparation procedures employed in monitoring antiepileptic drug concentrations using chromatographic techniques, encompassing both the method of obtaining and processing biological material and the specificity of the applied analytical technique. It includes various types of matrices—from plasma, serum, and saliva to DBSs—allowing for the assessment of drug pharmacokinetics in the human model and comparison of extraction method efficiency and determination of sensitivity and translational potential of the discussed chromatographic approaches.

**Table 2:** Description of sample preparation for monitoring drug concentration levels using chromatographic techniques in physiological fluids or tissues in the human model.

No.	Tissue/Body Fluid	Technique	Preparation	Determined Analytes	Ref.
1	Plasma, serum	LC	Deproteinisation: methanol in water, centrifugation, IS ([^2^H_5_]–phenobarbital), dilution	Phenobarbital	[54]
2	Plasma	HPLC	Deproteinisation: chilled acetonitrile, shaking, centrifugation, evaporation, reconstitution	Phenobarbital, phenytoin, primidone, CBZ, ethosuximide, lamotrigine, oxCBZ, rufinamide, zonisamide, lacosamide, LEV, felbamate and metabolites	[55]
3	Saliva	LC	Collection of 30 µL saliva on VAMS tips, drying 60 min, extraction: IS + methanol, shaking, sonication, evaporation under nitrogen, reconstitution with methanol	Perampanel	[49]
4	Plasma	LC	Plasma obtained after centrifugation of venous blood, deproteinisation with ACN, centrifugation, dilution, IS (diphenhydramine hydrochloride)	CBZ, lamotrigine, oxCBZ, 10–hydroxyCBZ, LEV, phenytoin, VPA, topiramate, phenobarbital, CBZ–E	[56]
5	Serum	LC	Deproteinisation: 250 µL methanol + 0.1% formic acid, mixing, centrifugation	oxCBZ, 10–monohydroxy derivative	[57]
6	Serum	UPLC	Deproteinisation: ACN, centrifugation, filtration, IS (propranolol)	Lacosamide, oxcarbazepine, lamotrigine	[58]
7	DBS	LC	Sample placed on filter card, extraction: methanol/ACN (3:1, *v*/*v*), 5 min ultrasonication, centrifugation, filtration	Lacosamide, lamotrigine, LEV, brivaracetam, phenytoin, gabapentin, pregabalin, primidone, rufinamide, zonisamide	[51]
8	DBS	GC	Carbon ink added to sample, acetone + 0.4% formic acid, HS analysis	VPA	[59]
9	Serum, plasma	LC	Sample dilution with IS:([^2^H_5_]–primidone), deproteinisation: methanol, mixing, centrifugation, dilution with mobile phase	Primidone	[60]
10	DPS	LC	Samples dried on Noviplex^®^ Plasma Prep Cards, extraction from dried plasma spot disc: (H_2_O:ACN, 50:50, *v*/*v*), addition of internal standard (VPA–d6, 10 µg/mL) and ACN, mixing, centrifugation	VPA	[61]
11	Plasma	HPLC	LLE: standard solution, reference solution, ethyl acetate, mixing, centrifugation, extraction repeated, evaporation under nitrogen, reconstitution in mobile phase, centrifugation	Perampanel, lamotrigine	[62]
12	Plasma, saliva	LC	Dilution, extraction: ACN with IS, mixing, centrifugation, reconstitution in mobile phase	LEV	[52]
13	Hair	LC	1 cm of cut hair, extraction: incubation in methanol, supernatant mixed with ACN and IS, centrifugation, evaporation, reconstitution in mobile phase	LEV	[52]
14	Plasma	HPLC	IS (clonazepam), extraction: dichloromethane, mixing, centrifugation, freezing of aqueous layer, decantation of organic layer, evaporation under nitrogen, reconstitution: (H_2_O:ACN, 3:1)	CBZ, CBZ–E	[53]
15	Serum, plasma	LC	IS ([^2^H12]–topiramate, deproteinisation) with methanol in H_2_O, centrifugation, dilution	Topiramate	[63]
16	Plasma	GC	Deproteinisation: trifluoroacetic acid, mixing, centrifugation, extraction: chloroform (AALLME)	VPA, 3–heptanone	[18]
17	Saliva	HPLC	Saliva collected using Super SAL^®^ device, LLE: IS (antipyrine), NaOH, mixing, portion of dichloromethane, centrifugation, transfer of organic layer, portion of dichloromethane, centrifugation, combination of organic layers, evaporation under nitrogen, reconstitution in Milli–Q H_2_O with ACN (80:20, *v*/*v*)	CBZ, CBZ–E, S–licarbazepine, lacosamide, LEV	[64]
18	Serum	GC	Extraction: BioSPME (conditioning: methanol/H_2_O (50:50, *v*/*v*)), IS, evaporation	VPA	[19]
19	Serum	UHPLC	Hydrolysis: KOH, incubation 100 °C 30 min, neutralisation with formic acid, LLE: M3^®^ buffer and acetone: ACN mixture (8:2, *v*/*v*), centrifugation	Cannabidiol and its metabolites	[65]

ACN—acetonitrile; HS—headspace; H_2_O—water; IS—internal standard; KOH—potassium hydroxide; LLE—liquid–liquid extraction; NaOH—sodium hydroxide; UHPLC—ultra–high–performance liquid chromatography; UPLC—ultra–performance liquid chromatography; VAMS—volumetric absorptive microsampling; VPA—valproic acid; VPA–d6—hexadeuterio valproic acid.

## 3. Chromatographic Techniques in the Diagnosis of Epilepsy

In the diagnosis and monitoring of epilepsy treatment, precise determination of metabolite concentrations and antiepileptic drugs and their metabolites in the patient’s body is crucial. Over the past 30 years, various chromatographic methods have been developed and optimised, enabling increasingly accurate and rapid determination of analytes in various biological matrices [66]. Chromatography constitutes a versatile analytical tool, enabling broad characterisation of studied compounds through the application of diverse detectors, analysers, and coupling with other analytical techniques. Depending on the physicochemical properties of analytes, selection of an appropriate chromatographic technique allows for effective separation and analysis of a wide spectrum of chemical compounds. The most frequently employed techniques are LC–MS and GC–MS. GC–MS is applied in the analysis of volatile and thermally stable metabolites, such as organic acids and low–molecular–weight lipids, often requiring derivatisation to increase analyte volatility whilst simultaneously ensuring high selectivity and sensitivity [13,67]. Conversely, LC–MS is used for the determination of non–volatile and thermally labile compounds, including amino acids, peptides, and nucleotides, employing reversed–phase liquid chromatography (RP–LC) for hydrophobic compounds or hydrophilic interaction chromatography (HILIC) for polar metabolites. Additionally, selection of the ionisation method in mass spectrometry enables adaptation of the analysis to the characteristics of the studied compounds, allowing for detection of both polar and non–polar analytes. Chromatography therefore has the potential to become a key tool in epilepsy diagnosis and treatment monitoring, and its further development may significantly improve the quality of patient care [68,69]. The utilisation of chromatographic techniques in epilepsy research demonstrates a clear tendency towards LC, which dominates particularly in the analysis of biomarkers and metabolites associated with disease pathogenesis. Nevertheless, GC also finds wide application, especially for the analysis of volatile organic compounds and in studies requiring high analytical resolution. GC is frequently coupled with mass spectrometry as a detection system (GC–MS), which constitutes a compatible and well–established combination deserving broader consideration in epilepsy research, particularly given its widespread availability in analytical laboratories. Both techniques are complementary, making it possible to obtain a more comprehensive picture of the biochemical processes occurring in patients with various forms of epilepsy. According to the conducted literature review, researchers more frequently selected LC. Among the employed techniques, ultra–performance liquid chromatography (UPLC), HPLC, and LC–MS/MS predominated, utilising both isocratic and gradient elution programmes [13,67].

The contemporary scientific literature documents the increasing application of chromatographic techniques in metabolomic research in epilepsy, demonstrating their growing role in the identification of specific epileptic biomarkers and monitoring of antiepileptic drugs. A particular advantage of these methods is their versatility and the ability to conduct both targeted analysis–directed at known predefined metabolites–and untargeted analysis, which enables comprehensive, simultaneous identification of numerous novel analytes without prior knowledge of their structure or occurrence. Among the presented methods, those utilising HRMS are particularly distinguished, as they allow for precise identification of compounds based on their accurate masses [42]. The headspace (HS) technique, successfully employed for the analysis of volatile compounds, is also of significant importance. Notably, an innovative method for analysis of valproic acid (VPA) in DBS using GC–MS with the application of ink auxiliary HS significantly improved determination sensitivity compared to the classical headspace gas chromatography coupled with mass spectrometry (HS–GC–MS) method without additives. Despite potential advantages in the context of drug concentration monitoring, the method relies on limited bioanalytical validation, the absence of detailed assessment of hematocrit effects, DBS stability, and matrix selectivity may significantly affect the reliability of determinations. Additional weaknesses of the study include a small clinical cohort size, lack of comparison with a reference method, and the use of a non–standard “ink–assisted” step, which limits inter–laboratory reproducibility. Consequently, it is not a clinical method used routinely but only an analytical method [59]. Additionally, the LC–MS^3^ method demonstrated high sensitivity, precision, and speed in therapeutic drug monitoring of oxcarbazepine (OXC) and its active metabolite, 10–monohydroxy derivative (MHD). While LC–MS^3^ enhances analytical sensitivity and selectivity, its clinical added value over established LC–MS/MS platforms has yet to be demonstrated in prospective, outcome–oriented studies. Although analytically robust, LC–MS^3^ should, therefore, be regarded at present as an advanced laboratory refinement rather than a clinically validated improvement in therapeutic decision making [57]. Chromatographic conditions can be selectively adapted and optimised to meet the needs of specific analyses. Although certain procedures may be further refined and validated, their transformation into routine diagnostic platforms or simplified point–of–care formats remains a long–term perspective requiring substantial technological, regulatory, and clinical validation. Transformation of the developed chromatographic methodology onto a test strip platform will enable implementation of advanced metabolic diagnostics in the format of rapid diagnostic systems, available for independent use by patients in home settings. However, it should be remembered that at this stage these are analytical methods and they are still far from clinical application [70].

### 3.1. Application of Chromatographic Techniques in Research on Metabolites Associated with Epilepsy and Recent Trends in the Diagnosis of Epilepsy

Recent trends in epilepsy diagnosis distinguish several principal metabolites with biomarker potential. Most frequently, epileptic seizures are associated with abnormal balance between neurotransmitters such as glutamate and γ–aminobutyric acid (GABA). Glutamate is the main excitatory neurotransmitter of the CNS, playing a crucial role in learning and memory processes. Excessive activation of glutamatergic receptors, such as the N–methyl–D–aspartate (NMDA) receptor or α–amino–3–hydroxy–5–methyl–4–isoxazolepropionic acid (AMPA) receptor, leads to oxidative stress and neuronal damage, contributing to epileptogenesis. Conversely, GABA serves as the main inhibitory neurotransmitter, and its deficiency results in excessive neuronal activity. In epilepsy, disturbances in dopaminergic and serotonergic pathways and reduced noradrenaline levels are also observed. Histamine participates in the regulation of neuronal excitability and inflammatory processes, whilst acetylcholine is involved in the regulation of cognitive functions. Disturbances of the cholinergic system are also observed in epilepsy [71]. In the scientific literature, reports may be found concerning choline metabolism disturbances observed in epilepsy. In research conducted by Lalwani et al. [31], alterations were observed in post–mortem brain tissue of patients with epilepsy, including disturbances in choline metabolism. The application of DI/LC–MS/MS combined with ^1^H–NMR represents a significant methodological advantage, enabling broad–profile analysis with high sensitivity and throughput. However, limited chromatographic separation resulting from the direct injection approach and the absence of detailed analytical validation reduce the certainty of quantitative interpretation and the translational potential of the method, while the fact that analyses were performed on post–mortem brain tissue makes their transfer to routine diagnostic tests and clinical applications practically impossible. The obtained metabolomic profiles may reflect both processes associated with epileptogenesis and the long–term impact of the disease on brain metabolism [31]. The analytical techniques employed were proton nuclear magnetic resonance spectroscopy (^1^H–NMR) and direct injection/liquid chromatography coupled with tandem mass spectrometry (DI/LC–MS/MS). Samples collected from the frontal cortex of patients and a control group were analysed, identifying metabolites with altered concentrations, including O–acetylcholine and adenosine monophosphate (AMP). The results indicate disruptions in choline metabolism and beta–oxidation of fatty acids, emphasising the critical role of oxidative stress and energy disturbances in epilepsy pathogenesis [31].

**Figure 2:**
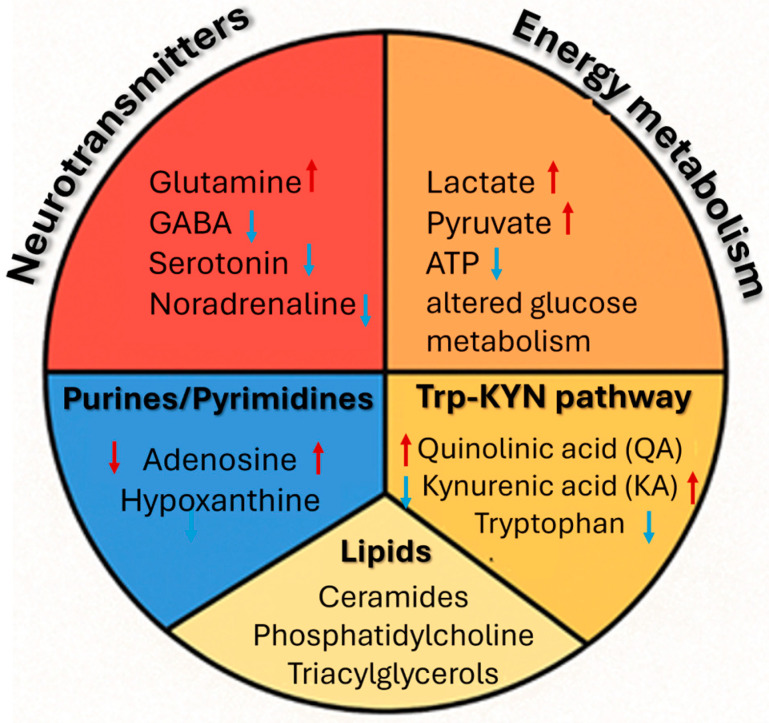
Presents the main classes of metabolites engaged in the pathophysiology of epilepsy (neurotransmitters, energy metabolites, tryptophan–kynurenine pathway compounds, purines, pyrimidines, and lipids), which constitute the most frequently analysed groups in chromatographic studies. The direction of changes in their concentrations is indicated by arrows, reflecting the characteristic metabolic disturbances described in patients and in animal models.

Figure 2 Classes of principal metabolites associated with the pathophysiology of epilepsy together with the direction of changes in their concentrations reported in clinical and experimental studies.

Epilepsy is also associated with significant alterations in the brain’s energy processes, which may be both a cause and consequence of the disorder. Glucose constitutes the primary energy source; however, under epileptic conditions, changes in its metabolism are observed, including increased lactate production by astrocytes. Astrocytes play a key role in regulating ionic balance and neuronal energy metabolism, which may lead to increased neuronal excitability and decreased glutamate uptake, thereby intensifying epileptic phenomena [72]. Adenosine, a purine metabolite, acts as an endogenous antiepileptic agent via A1 receptors, reducing seizure activity and regulating brain energy consumption. In epilepsy, disturbances in its metabolism occur, primarily as a result of excessive activity of the enzyme adenosine kinase (ADK), which reduces adenosine levels and increases neuronal excitability. Regulation of its concentration represents a potential therapeutic target in epilepsy treatment [73]. Brain–nourishing metabolites, including glucose and taurine, were also investigated by Akiyama et al. [74] and Guo et al. [75]. Akiyama, utilising GC–MS/MS and LC–MS/MS, analysed the metabolite profile in urine of children with newly diagnosed epilepsy, identifying reduced taurine levels as a significant marker differentiating patients from the control group. Guo, conversely, focused on plasma samples from children with drug–resistant epilepsy. The study demonstrated disturbances in glucose metabolism, indicating significant alterations in the brain’s energy economy. Both studies employed LC–MS/MS, enabling detection of changes in energy metabolite levels, which emphasises their potential role in epilepsy pathogenesis and their possible utilisation as diagnostic biomarkers [74,75]. Recently, research concerning the microbiota–a complex ecosystem of microorganisms colonising specific environments in the human body–has attracted significant scientific interest. This diverse community of bacteria, fungi, viruses, and archaea performs crucial functions in maintaining health by supporting digestive processes, producing vitamins, and regulating the immune system. These studies are of particular interest in the context of epilepsy, as they have demonstrated significant alterations in the structure of the gut microbiota and metabolite profiles in patients with epilepsy. In research conducted by Zhou et al. [76], it was observed that individuals with epilepsy exhibit increased quantities of Proteobacteria and Actinobacteria, whilst bacteria from the *Firmicutes phylum* are decreased. Significant differences are also observed in the concentrations of amino acids, lipids, and carbohydrates between patients and the control group, as well as reduced levels of tryptophan and kynurenine. Certain bacterial groups (e.g., *Ruminococcus*, *Bacteroides,* and *Veillonella*) have also been associated with tryptophan content. To conduct targeted metabolomic studies, ultra–high–performance liquid chromatography coupled with tandem mass spectrometry (UHPLC–MS/MS) was applied. Tryptophan and kynurenine play a fundamental role in central nervous system function, which has been confirmed in a series of studies on the pathophysiology of temporal lobe epilepsy. Despite the application of integrated macrogenomic and metabolomic analysis, the study is exploratory in nature and does not include full analytical validation or replication in independent cohorts, which limits the interpretation of the proposed metabolites as biomarkers. Additional limitations include the cross–sectional design, small sample size, and lack of control for confounding factors, such as antiepileptic treatment, diet, or inter–individual microbiota variability. Consequently, the obtained results should be regarded as hypothesis–generating biological observations that do not permit direct diagnostic application. Research conducted by Dey et al. [45] provided significant evidence in this regard, utilising analytical techniques for comprehensive assessment of the kynurenine pathway. The researchers employed HPLC with fluorescence detection to determine the concentrations of tryptophan, kynurenine, and kynurenic acid whilst using LC–MS/MS to measure quinolinic acid, glutamate, and GABA. The research material consisted of hippocampal tissue samples obtained from 55 patients with mesial temporal lobe epilepsy with hippocampal sclerosis (MTLE–HS) who underwent resection, 15 autopsy samples, and 20 control samples obtained during brain tumour surgery. The results of these analyses suggest that disturbances in the tryptophan–kynurenine metabolic pathway may constitute a key factor contributing to enhanced glutamatergic activity and pathological neuronal hyperactivity in epilepsy. Despite the application of advanced analytical and electrophysiological methods and direct analysis in human hippocampal tissue, which constitutes significant mechanistic value of the study, the work is cross–sectional in nature and does not include biomarker validation in body fluids or longitudinal analysis. Limitations include, among others, a selective population of patients with drug–resistant epilepsy and lack of control for the effect of antiepileptic treatment on kynurenine pathway metabolism. Consequently, the obtained results cannot be regarded as a diagnostic tool or marker of therapeutic efficacy. However, the obtained metabolomic profiles may reflect both processes associated with epileptogenesis and the long–term impact of the disease on brain metabolism [45]. Further research confirming the involvement of the gut–brain axis in epilepsy pathophysiology was conducted by Ouyang et al. [77]. The results suggest that specific bacteria may serve as biomarkers and therapeutic targets in certain forms of epilepsy. Bacteria from the *Veillonellaceae* family were associated with increased risk of childhood absence epilepsy. The class *Melainabacteria* was found to reduce the risk of generalised epilepsy, whilst the class *Betaproteobacteria* and order *Burkholderiales* influenced juvenile myoclonic epilepsy. The applied methodology may enable a more comprehensive understanding of epileptogenesis [77]. In research conducted by Meier et al. [42], two different chromatographic columns–HILIC and C18–were employed for analysis of a wide spectrum of energy metabolites, enabling effective separation of both polar and non–polar compounds. Through this approach, a comprehensive metabolomic profile was obtained in which disturbances in the levels of tryptophan, glutamine, adenosine, cholesterol, and other key metabolites were observed. The significance of this study is further emphasised by the employment of HRMS, which enabled observation of dynamic changes in the hippocampal metabolic profile at various stages of epileptogenesis. As early as 3 h after induction of status epilepticus (SE), an increase in tryptophan and N–acetylornithine levels was found, suggesting activation of neuroinflammatory pathways and disturbances in neurotransmission. After one week, a significant decrease in the levels of over 170 metabolites occurred, including N–acetylaspartate (NAA), glutamine, adenosine, and cholesterol, indicating neuronal damage, mitochondrial dysfunction, and increased neuronal excitability. Two weeks after SE, these changes persisted or intensified. The application of UPLC–HRMS in an untargeted metabolomics approach represents a significant methodological advantage, enabling high–resolution analysis of metabolic changes in the hippocampus at different stages of epileptogenesis. However, the absence of full quantitative validation, identification of some metabolites only at the putative level, and the fact that analyses were performed on brain tissue from an animal model significantly limit the possibility of translating the method to routine diagnostic testing and clinical applications. The obtained metabolic profiles may reflect both the process of epileptogenesis and the long–term impact of the disease on brain metabolism [42]. A detailed literature review is presented in Table 3, where the focus was placed on the chromatographic techniques employed, with consideration of the detectors and temperature/elution programmes used in the research on the search for novel diagnostic markers.

**Table 3:** Application of analytical methods employing chromatographic techniques in search of markers of drug–resistant epilepsy in body fluids and tissues in humans.

**GC**									
**No.**	**Type of Sample**	**Analytical Technique**	**Column**	**Temperature Programme**			**Detector**	**Determined Metabolites/Groups of Metabolites**	**Ref.**
1	Urine	GC–MS/MS	BPX–5 capillary	60 °C (2 min) → ↑15 °C/min → 330 °C (3 min)			QQQ	172 metabolites: amino acids, organic acids, fatty acids, carbohydrates, nitrogenous compounds and polyamines	[74]
2	CSF	GC–MS	BPX–5 capillary	60 °C (2 min) → ↑15 °C/min → 330 °C (3 min)			QQQ	56 metabolites including: glycine, xylose, ketoisocaproic acid	[78]
3	DBS	GC–MS	DB–5MS capillary	50 °C (1 min) → ↑20 °C/min → 330 °C (5 min)			Triaxial	Glutamine, pyruvic acid, L–serine, oxalic acid, caprylic acid, palmitic acid	[79]
4	Plasma	GC–MS	DB–5MS capillary	60 °C (2 min) → 5 °C/min → 285 °C (2 min)			Q	Phosphate, proline, lactic acid, alanine, glutamate, hexadecanoic acid	[20]
5	Plasma	GC–TOF–MS	DB–5MS capillary	70 °C (2 min) → ↑30 °C/min → 320 °C (2 min)			TOF–MS	Proline, glutamate, phenylalanine, methionine, lysine, tryptophan, citric acid, uric acid, cholesterol, palmitate, glucose, myo–inositol, creatinine	[80]
6	Serum	GC–MS	DB–5MS capillary	60 °C (2 min) → ↑5 °C/min → 285 °C (2 min)			Q	Amino acids: glutamate, proline, asparagine, cysteine. Fatty acids: palmitic acid, linoleic acid, stearic acid, elaidic acid	[33]
7	CSF	GC–MS/MS	CP–SIL 8 CB	80 °C (2 min) → ↑15 °C/min → 330 °C (6 min)			QQQ	2–Ketoglutaric acid, pyridoxamine, tyrosine, 1,5–anhydroglucitol, 2–aminobutyric acid, 2–ketoisocaproic acid, 2–propyl–5–hydroxypentanoic acid, 4–hydroxyproline, acetylglycine, methionine, N–acetylserine, serine	[81]
8	Urine/ plasma	GC–MS/MS	CP–SIL 8 CB	80 °C (2 min) → ↑15 °C/min → 330 °C (6 min)			QQQ	3–Hydroxybutyrate, acetoacetate, 2–hydroxybutyrate, 3–hydroxyisobutyrate, acetylglycine, decanoic acid, octanoic acid, isoleucine, adipic acid, uric acid, glyoxylic acid, citric acid, tartaric acid, glucosamine, galactose, mannitol, N–acetyl–lysine, 2–aminopimelate, 3–hydroxyanthranilate	[82]
**LC**									
**No.**	**Type of Sample**	**Analytical Technique**	**Detector**	**Column**	**Column Temp.**	**Mobile Phase**	**Elution Programme**	**Determined Analytes**	**Ref.**
1	Plasma	HILIC–LC–MS/MS, ESI	Triple TOF	Acquity UPLC BEH Amide	40	A: H_2_O + 25 mM NH_4_OAc + 25 mM NH_4_OH; B: 100% ACN	B%: 1 min–95%; 14–65%; 16–40%; 18–40%; 18.1–95%; 23–95%	Polar metabolites: amino acids (glutamine, glycine), organic acids (citric acid), energy metabolites (glucose), amides	[75]
2	Plasma	HILIC–LC–MS/MS, ESI	Triple TOF	Acquity UPLC BEH Amide	40	A: H_2_O + 25 mM NH_4_OAc + 25 mM NH_4_OH; B: 100% ACN	B%: 1 min–95%; 14–65%; 16–40%; 18–40%; 18.1–95%; 23–95%	Negatively charged metabolites: fatty acids (linoleic acid), organic acids (2–oxoglutarate), bile acids, some energy metabolites	[75]
3	Plasma	LC–MS/MS	QQQ–MS/MS	Supelco Discovery HS F5 HPLC	30	A: H_2_O + 3.7% acetic acid + 0.01% HFBA; B: ACN + 0.1% formic acid	100% A (2 min) → 5% B (3 min) → 50% B (4 min) → 100% B (1 min) → 100% B (1 min) → 0% B (0.5 min) → re–equilibration (4.5 min)	Guanidinoacetate, creatine, sulphocysteine, pipecolic acid, pyridoxal–5–phosphate, pyridoxal, pyridoxine, pyridoxamine, pyridoxic acid, proline	[83]
4	Urine	LC–MS/MS	QQQ–MS/MS	Supelco Discovery HS F5 HPLC	30	A: H_2_O + 3.7% acetic acid + 0.01% HFBA; B: ACN + 0.1% formic acid	100% A (2 min) → 5% B (3 min) → 50% B (4 min) → 100% B (1 min) → 100% B (1 min) → 0% B (0.5 min) → re–equilibration (4.5 min)	Guanidinoacetate, creatine, sulphocysteine, pipecolic acid, pyridoxal–5–phosphate, pyridoxal, pyridoxine, pyridoxamine, pyridoxic acid, alpha–aminoadipic semialdehyde, piperideine–6–carboxylate	[83]
5	Plasma/urine	UPLC–MS/MS	QQQ–MS/MS	BEH–C18	40	A: H_2_O + 0.1% formic acid; Methanol + 0.1% formic acid	30% B (0 min, 0.33 mL/min) → 70% B (2 min, 0.25 mL/min) → 95% B (4 min, 0.33 mL/min) → 30% B (5 min, 0.33 mL/min) → 30% B (7 min, 0.33 mL/min)	α–Aminoadipic semialdehyde, piperideine–6–carboxylate, pipecolic acid	[32]
6	CSF	UPLC–MS/MS	TQ–S QQQ–MS	Waters BEH C18	30	A: H_2_O + 0.1% formic acid, B: methanol	0–0.1 min → 5% B/2.7 min → 35% B/2.9–3.25 min → 95% B/ 3.4–4.7 min → 5% B	Pyridoxal–5′–phosphate, pyridoxal, pyridoxine, pyridoxamine, pyridoxic acid	[35]
7	Plasma/CSF	RP–LC	HRMS +	Hypersil GOLD C18	30	A (100% H_2_O + 0.1% formic acid) B (100% ACN + 0.1% formic acid)	5% B (2 min) → 100% B (11 min) → 100% B (12.5 min) → return to start	Succinic acid, mevalonic acid, kynurenic acid, pyruvate, hypoxanthine, theobromine, caffeine, uracil	[84]
8	Plasma/CSF	HILIC	HRMS −	Sequant ZIC–pHILIC	15	A (10 mM ammonium carbonate, pH 10.5) B (ACN)	80% B (2 min) → 40% B (10 min) → 0% B (5 min) → return to start	Glutamine, arginine, asparagine, N–acetylaspartylglutamate, tryptophan, adenosine, quinolinic acid, uridine	[84]
9	CSF (microdialysate)	HPLC	FLD	3IM Phase II ODS	Not reported	0.1 M acetic acid in 37% ACN solution	Isocratically	Glutamate, glutamine	[28]
10	CSF (microdialysate)	HPLC	ECD	3IM Phase II ODS	Not reported	0.1 M acetic acid in 37% ACN solution	Isocratically	GABA	[28]
11	Plasma/CSF	UPLC–MS/MS	QTRAP	ACQUITY HSS T3	40	A: 0.1% formic acid in H_2_O B: 0.1% formic acid in ACN	(A:B) 0–0.5 min (98:2) → 11.5 min (70:30) → 11.51 min (10:90) → 13 min (10:90) → 13.1 min (98:2) → 16 min (98:2)	33 Dipeptides and amino acids	[36]
12	Plasma/urine/ DBS	LC–MS/MS	QQQ	ACQUITY BEH C18	40	A: 0.1% formic acid in H_2_O B: 0.1% formic acid in methanol	0 min (10% B, 0.4 mL/min) → 1.5 min (45% B, 0.3 mL/min) → 2.5 min (75% B, 0.3 mL/min) → 3 min (95% B, 0.3 mL/min) → 4–5 min (10% B, 0.3 mL/min)	α–Aminoadipic semialdehyde, α–aminoadipic acid, pipecolic acid	[40]
13	DBS	UPLC–MS/MS	QQQ–MS MRM	Waters Acquity UPLC HSS T3	Not reported	A: 3.7% acetic acid in H_2_O + 0.01% heptafluorobutyric acid B: 100% methanol	(%A | %B) 0.00 min (97.5:2.5) → 0.40 min (97.5:2.5) → 3.75 min (50:50) → 4.25 min (0.1:99.9) → 5.00 min (97.5:2.5) → 6.50 min (97.5:2.5)	Pyridoxal–5′–phosphate, pyridoxine–5′–phosphate, pyridoxamine–5′–phosphate	[44]
14	Hippocampus	HPLC	FLD	Phenomenex C18	37	50 mM acetic acid, 100 mM zinc acetate, 3% ACN	Isocratically	Tryptophan, kynurenine, kynurenic acid, PLP	[45]
15	Hippocampus	LC–MS/MS	QQQ–MS	Waters C18	Not reported	A: methanol B: 0.05% formic acid in H_2_O + 1 mM heptafluorobutyric acid	Not reported	Quinolinic acid, glutamate, GABA	[45]
16	CSF	HPLC	FLD	Waters Atlantis T3	35	A: 60 mM phosphate buffer with EDTA, B: 100% ACN, C: Ultrapure H_2_O	(98.6% A: 1.4% B: 0% C) → 3.5 min (98.6% A: 1.4% B: 0% C) → 7.0 min (94% A: 6% B: 0% C) → 7.1 min (0% A: 60% B: 40% C) → 10.0 min (98.6% A: 1.4% B: 0% C) → 20.0 min (98.6% A: 1.4% B: 0% C)	Pyridoxal 5′–phosphate, pyridoxal, 4–pyridoxic acid	[46]
17	Serum	LC–MS	Q–Orbitrap FTMS	ACQ UITY UPLC HSS T3	40	A: 95% H_2_O + 5% ACN (containing 0.1% formic acid) B: 47.5% ACN + 47.5% isopropanol + 5% H_2_O (containing 0.1% formic acid)	Not reported	Malathion monocarboxylic acid, serylproline, asparagylthreonine, 7–methyl–3–oxo–6–octenoyl–coenzyme A, aspartyl–phenylalanine, phenylalanylphenylalanine, artonol B	[41]
18	Urine	LC-MS/MS	QQQ–S	Not reported	Not reported	MxP^®^ Quant 500 measurement kit	MxP^®^ Quant 500 measurement kit	Taurine, hexoses, quinolinic acid, N–acetylneuraminic acid, catechol	[74]
19	Urine/ plasma	LC-MS/MS	QQQ	Not reported	Not reported	MxP^®^ Quant 500 measurement kit	MxP^®^ Quant 500 measurement kit	Glutamate, 2–aminobutyrate, 3–aminobutyrate, acetyl–L–carnitine, 3–hydroxybutyrylcarnitine, stearoylcarnitine, diglycerides, triglycerides, ceramides, phosphatidylcholine, cysteine, cystine, homoarginine, lysophosphatidylcholine, sphingomyelins	[82]

ACN—acetonitrile; DBS—dried blood spot; ECD—electron capture detector; EDTA—ethylenediaminetetraacetic acid; FLD—fluorescence detector; H_2_O—water; HILIC–LC–MS/MS, ESI—hydrophilic interaction liquid chromatography–tandem mass spectrometry with electrospray ionisation in positive mode; HILIC–LC–MS/MS, ESI—hydrophilic interaction liquid chromatography–tandem mass spectrometry with electrospray ionisation in negative mode; NH_4_OAc—ammonium acetate; NH_4_OH—ammonium hydroxide; PLP—pyridoxal 5′–phosphate; Q—single quadrupole detector; Q–Orbitrap FTMS—quadrupole–orbitrap Fourier transform mass spectrometry; QQQ—triple quadrupole detector; QQQ–MS—triple quadrupole mass spectrometry detector; QQQ–MS MRM—triple quadrupole mass spectrometry detector in multiple reaction monitoring mode; QQQ–MS/MS—triple quadrupole tandem mass spectrometry detector; QTRAP—quadrupole linear ion trap mass spectrometer detector; TOF—time–of–flight analyser; TOF–MS—mass spectrometry with time–of–flight analyser; UPLC–MS/MS—ultra–performance liquid chromatography–tandem mass spectrometry.

Extensive research has also been conducted on neurotransmitters in animal models, utilising high–performance liquid chromatography with electrochemical detection (HPLC–ECD) for metabolite analysis. In the study, glutamate and GABA, as well as dopamine and serotonin, were determined in samples collected from Swiss albino mice, divided into study groups of 6 individuals each. It was observed that in mice subjected to pentylenetetrazole kindling, constituting a model of epilepsy, glutamate levels increased along with a decrease in GABA and serotonin, and an increase in oxidative stress. Choline administered to one of the study groups restored neurotransmitter balance, improving cognitive functions and additionally reducing depressive symptoms. The results of the analysis provided significant information regarding changes in neurotransmitter concentrations in the brain, allowing for a better understanding of their role in mechanisms associated with epilepsy. The application of HPLC for brain tissue analysis serves a supporting role in relation to behavioural and pharmacological analyses. The part of the study utilising these tissues is practically clinically inapplicable in humans. A valuable extension of the study would be the transfer of the method for metabolite determination in serum to liquid chromatography, as this would eliminate the need for derivatisation and enable the application of clinical matrices such as plasma or cerebrospinal fluid [21]. In subsequent research conducted on a rat model of temporal lobe epilepsy, alterations in lipid, purine, and sterol metabolism were observed, including a significant reduction in 25–hydroxyvitamin D_3_ levels. The study employed an untargeted metabolomics technique based on LC–MS and high–performance liquid chromatography coupled with tandem mass spectrometry (HPLC–MS/MS) for analysis of metabolite changes in the plasma and hippocampus of rats with a temporal lobe epilepsy model induced by kainic acid. Significant alterations were observed in lipid metabolism (phosphatidylcholine, ceramides, and triacylglycerols), purines (adenosine and hypoxanthine), amino acids (proline and citrulline), and vitamin D_3_ and its derivatives (including a decrease in 25–hydroxyvitamin D_3_ in plasma in the acute and latent phases). The direction of changes depended on the disease phase: in plasma, decreases predominated in the acute and chronic phases, whilst in the hippocampus, increases in metabolites were most evident in the latent phase [34]. Fujita et al. (2019) [39] conducted analysis of volatile organic compounds (VOCs) in urine samples in an animal model of temporal lobe epilepsy induced by amygdala kindling, using SPME in combination with GC–MS. The study demonstrated significant differentiation of the metabolomic profile between the experimental and control groups, identifying 15 potential biomarkers, including ketone compounds (2–butanone and 2–pentanone), sulphur compounds (dimethyl disulphide and methanethiol), amines (trimethylamine), and heterocyclic compounds (2–acetylpyrroline). Bidirectional changes in the levels of these metabolites were observed: an increase in ketone compound concentrations and a decrease in sulphur compound and 2–heptanone concentrations. The obtained results suggest the potential diagnostic value of the VOC profile as biomarkers in the identification and monitoring of drug–resistant forms of epilepsy. The applied technique represents a significant methodological innovation, as it allows for the analysis of volatile metabolites with minimal sample preparation and reduction of extraction steps, which significantly simplifies the chromatographic workflow. At the same time, the study is preliminary in nature, and the absence of full analytical validation indicates that this approach requires further refinement, standardisation, and confirmation in subsequent analyses. Despite these limitations, the method represents an innovative exploratory approach that requires further validation before any clinical application can be considered [39]. A detailed literature review is presented in Table 4, where the focus was placed on the application of analytical methods employing chromatographic techniques in research on the search for markers in animal models, with particular consideration of studies conducted on mice and rats.

**Figure 3:**
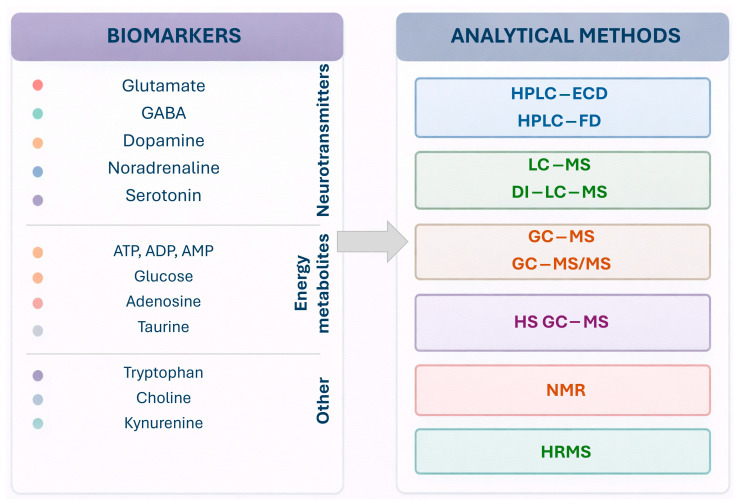
The most important biomarkers associated with epilepsy and analytical methods suitable for their identification and quantification.

Figure 3 presents a summary of the most important biomarkers associated with epilepsy and a wide range of analytical methods suitable for their identification.

### 3.2. Monitoring of Drugs and Their Metabolites in Human and Animal Tissues and Body Fluids

Antiepileptic drugs (AEDs), previously termed anticonvulsants, constitute the foundation of symptomatic treatment of epilepsy. Due to their diverse mechanisms of action, the majority of AEDs do not demonstrate efficacy against all seizure types, emphasising the importance of appropriate therapy selection. Consequently, novel treatment strategies are being developed, directed at specific epilepsy aetiologies and focused on individual patient predispositions. To date, over 30 antiepileptic drugs have been approved for clinical use; however, approximately 30% of patients still exhibit pharmacoresistance. An important aspect of treatment is the adverse effect profile. Chronic use of antiepileptic drugs may lead to tolerance issues, drug interactions, withdrawal symptoms, and increased economic burden, emphasising the need for further research on more effective and safer methods of epilepsy therapy. Therapeutic drug monitoring should be common practice to adjust doses and minimise the risk of adverse effects in individual patients [89,90,91]. Carbamazepine (CBZ) and lamotrigine are recommended as first–line drugs in the treatment of partial seizures [92]. Phenobarbital and phenytoin, although belonging to older drugs, still find application, particularly in lower–income countries, due to their availability and low cost [93]. Sodium valproate is preferred in the treatment of generalised tonic–clonic seizures, whilst levetiracetam (LEV) may constitute an alternative to CBZ and lamotrigine in partial seizures and to sodium valproate in generalised seizures. Third–generation drugs, such as lacosamide, eslicarbazepine, perampanel, and brivaracetam, are characterised by better tolerability and fewer interactions with other drugs, making them an attractive therapeutic option, particularly in patients resistant to standard treatment [94,95,96]. In research conducted by Milosheska and Roskar [55], HPLC with UV detection was utilised to determine AEDs and their metabolites in the plasma of patients with epilepsy. Twelve substances were analysed, including CBZ, phenobarbital, phenytoin, oxcarbazepine (OXC), zonisamide, and lamotrigine. The presented method was validated, demonstrating linearity (R^2^ > 0.99), precision and accuracy (CV and bias ≤ ±15%), and analyte recovery stability (≥85%). The application of gradient HPLC–UV with a Phenyl–Hexyl column represents a strength of the work, enabling simultaneous separation of multiple antiepileptic drugs through enhanced selectivity resulting from π–π interactions. Despite validation in accordance with FDA guidelines, the use of UV detection limits analytical specificity and the range of analysed compounds compared to LC–MS/MS techniques. Consequently, the developed chromatographic method may be useful in routine laboratory determinations; however, its implementation potential remains limited by lower sensitivity and selectivity of detection [55]. Another example of the application of chromatographic techniques is monitoring the concentration of an AED (perampanel) in the saliva of individuals with epilepsy. In the study, LC–MS/MS was employed to determine perampanel in patients’ saliva, utilising the volumetric absorptive microsampling (VAMS) technique. VAMS is a technique for collecting small, precise volumes of biological fluids (primarily blood) using a special hydrophilic material with a sponge–like structure. The device absorbs a precisely defined volume of fluid (10–30 μL), independent of haematocrit, after which the sample is dried and subjected to extraction prior to instrumental analysis. VAMS is distinguished by low invasiveness, simplicity of use, sample stability at room temperature, and quantitative accuracy. Validation using this extraction technique demonstrated high precision, accuracy, and stability, confirming the utility of this method in therapeutic drug monitoring of perampanel concentrations, which constitutes a non–invasive alternative to plasma determinations [49]. An interesting approach to non–invasive drug monitoring and patient self–management of therapy in epilepsy is characterised by research using dried blood spots (DBSs) [50]. In the study, an LC–MS/MS method was developed and validated, enabling quantitative determination of 22 antiepileptic drugs and five of their metabolites in DBS samples, with results compared to serum analysis. An analysis of 282 blood samples demonstrated significant differences between DBSs and serum: lamotrigine levels were 14% higher in serum, LEV levels were 15% lower in serum, whilst brivaracetam exhibited comparable values. It is worthwhile to develop methods utilising DBSs so that they may be suitable for therapeutic drug monitoring of AED concentrations, constituting a non–invasive alternative to traditional determinations in serum or other physiological fluids. The developed chromatographic approach can be considered a promising tool for pharmacotherapy monitoring, which requires further standardisation and consideration of matrix variability before full clinical implementation [51].

A detailed literature review concerning chromatographic techniques employed in the monitoring of drugs and their metabolites in patient samples of physiological fluids and human tissues, together with detailed consideration of the detectors utilised and the temperature and elution programmes applied, is presented in Table 5.

Research on human tissues for determination of metabolites characteristic of epilepsy is practically unfeasible because it would require performing highly invasive biopsies. For this reason, these studies are conducted on animals. Animal models are frequently utilised as surrogates for humans and are crucial in metabolomics, enabling access to tissue–specific metabolism that is often impossible to investigate in humans due to ethical limitations [103]. An exemplary study utilising animal tissues is the analysis of adenosine triphosphate (ATP) metabolism and its derivatives in the hippocampus of rats with temporal lobe epilepsy induced by pilocarpine. In the study, microdialysis in combination with HPLC and fluorescence detection was employed, enabling precise determination of ATP, adenosine diphosphate (ADP), adenosine monophosphate (AMP), and adenosine levels. The experiment included Wistar rats divided into acute, chronic, and control groups, with metabolites analysed at various stages of epileptogenesis. The results demonstrated that ATP may act in an excitatory manner in the acute phase, whilst adenosine performs a neuroprotective function. In the chronic phase, disturbances in ATP metabolism may promote epileptic seizures, suggesting a crucial role of purine transformations in the mechanisms of epileptogenesis. The research is practically impossible to transfer to human cases due to the high level of interference and risks associated with brain tissue analysis and microdialysis. However, it provides a promising picture of biomarkers associated with epileptogenesis and chronic disease [37].

### 3.3. Extracellular Vesicle Metabolomics and Metabolomics–Guided Identification of Enzymatic Targets in Epilepsy

Extracellular vesicles (EVs), including exosomes and microvesicles, have recently emerged as a highly promising source of disease biomarkers in neurological disorders due to their ability to cross the blood–brain barrier (BBB) and to reflect molecular processes occurring in the central nervous system [104,105]. EVs are lipid bilayer, enclosed particles released by virtually all cell types, including neurons, astrocytes, microglia and endothelial cells, and are abundantly present in biological fluids such as plasma, serum, cerebrospinal fluid and saliva [106]. Their unique biological properties make them particularly attractive for translational research aimed at identifying minimally invasive biomarkers of epilepsy. Notably, the metabolite classes summarised in Figure 2, particularly neurotransmitters and intermediates of energy metabolism, are well represented within EV cargo, reinforcing the relevance of EV–based metabolomics as a future extension of the conceptual framework presented in this review.

Importantly, EVs carry a complex and selectively enriched molecular cargo comprising proteins, nucleic acids, lipids and low–molecular–weight metabolites, thereby representing a biologically protected compartment that reflects the intracellular metabolic state of the cell of origin [104]. In contrast to conventional bulk biofluids, EV–based metabolomics provides enhanced molecular specificity, reduced background noise and improved stability of labile metabolites, which are often prone to rapid degradation in circulation [107]. Recent studies have demonstrated that neurotransmitters, energy metabolites and lipid mediators involved in neuronal signalling and neuroinflammation can be detected in EV fractions isolated from blood and cerebrospinal fluid [107,108]. This opens new perspectives for non–invasive monitoring of brain–derived metabolic alterations associated with epileptogenesis, seizure activity and pharmacological response. In particular, EV–associated metabolites related to glutamatergic and GABAergic signalling, purine metabolism, mitochondrial function, and oxidative stress may constitute a novel class of circulating biomarkers with improved diagnostic and prognostic performance compared to free circulating metabolites [109].

From an analytical perspective, the integration of EV isolation strategies—such as ultracentrifugation, size–exclusion chromatography, immunoaffinity capture or emerging microfluidic platforms—with high–resolution chromatographic techniques (LC–MS/MS, UPLC–HRMS, and GC–MS) enables both targeted and untargeted profiling of EV metabolomes with high sensitivity and broad molecular coverage [107]. Nevertheless, significant methodological challenges remain, including standardisation of EV isolation protocols, normalisation strategies, limited sample amounts and the lack of harmonised validation frameworks specifically adapted to EV–based metabolomics [106,107]. Addressing these limitations is essential for future clinical implementation of EV–derived metabolic biomarkers in epilepsy.

Beyond the identification of altered metabolite profiles, an important translational dimension of metabolomic research in epilepsy lies in the metabolomics–guided discovery of enzymatic biomarkers and therapeutic targets. Since metabolite concentrations represent downstream readouts of enzymatic activity, systematic metabolomic profiling enables the identification of dysregulated biochemical pathways and the pinpointing of rate–limiting enzymes that drive pathological processes [109]. In epilepsy, disturbances in purine metabolism, amino acid neurotransmission, the tryptophan–kynurenine pathway and energy metabolism can be directly linked to specific enzymatic systems, including ADK, glutamate decarboxylase (GAD), monoamine oxidases (MAO), kynurenine aminotransferases (KATs) and enzymes of mitochondrial oxidative phosphorylation [110,111].

Importantly, these enzymes not only serve as mechanistic biomarkers reflecting disease activity, but also constitute rational therapeutic targets, as modulation of their activity may restore metabolic homeostasis and neuronal excitability [109,112]. Excessive ADK activity, leading to adenosine deficiency—one of the key endogenous anticonvulsant mechanisms—has been strongly implicated in epileptogenesis and seizure susceptibility [110]. Similarly, alterations in the activity of enzymes of the kynurenine pathway may shift the balance between neuroprotective and neurotoxic metabolites, thereby modulating excitatory neurotransmission and neuroinflammatory processes [112]. Dysregulation of GAD activity directly affects GABA synthesis, contributing to excitatory–inhibitory imbalance, while EV–associated signatures of mitochondrial dysfunction, such as altered acylcarnitine profiles, point towards impaired energy metabolism in drug–resistant epilepsy. Importantly, EV–associated metabolite patterns may provide indirect yet functionally relevant readouts of intracellular enzymatic activity, enabling simultaneous assessment of metabolic dysregulation and its enzymatic drivers.

From a systems biology perspective, integration of EV–based metabolomics with enzymatic network analysis, pathway modelling and complementary omics approaches (proteomics and transcriptomics) enables identification of metabolic bottlenecks and druggable nodes, facilitating a transition from descriptive metabolite signatures towards mechanism–driven therapeutic strategies [109]. Consequently, future studies should focus on the systematic characterisation of EV–derived metabolomic signatures in well–defined epilepsy cohorts, longitudinal assessment of EV metabolite dynamics during disease progression and therapy, and enzyme–centric validation using functional and activity–based assays. Such an integrated, multi–omics approach has the potential to translate metabolomic findings into clinically actionable biomarkers and targeted therapeutic interventions, advancing precision medicine in epileptology [112].

## 4. Validation and Quality Control

Validation of chromatographic methods in metabolomic analyses constitutes an indispensable element of every reliable analytical workflow, ensuring the credibility, reproducibility, and accuracy of the results obtained. To ensure the analytical reliability and interpretability of metabolomic data, both targeted and untargeted approaches require rigorous validation and quality control procedures. The validation process should comply with the guidelines of recognised regulatory institutions, such as the International Council for Harmonisation (ICH), the European Medicines Agency (EMA), and the United States Food and Drug Administration (FDA). Reference documents, including ICH Q2(R2) Validation of Analytical Procedures and FDA guidelines for bioanalytical method validation, define a comprehensive framework of analytical performance characteristics that should be evaluated during method development and implementation, taking into account the intended purpose of the analytical procedure. In targeted metabolomics, validation is performed according to well–defined regulatory criteria and focuses primarily on the assessment of analytical performance characteristics, including selectivity, linearity, validated range, limit of detection (LOD), limit of quantification (LOQ), accuracy, precision (encompassing repeatability and intermediate precision), recovery, and analyte stability. The validated range is defined as the concentration interval over which acceptable linearity, accuracy, and precision are demonstrated. Together, these parameters ensure that quantitative measurements are robust, reproducible, and suitable for reliable comparison between samples and study groups. In addition, method robustness—defined as the ability of the analytical procedure to remain unaffected by small, deliberate variations in method parameters—represents an important supplementary performance characteristic in accordance with ICH Q2(R2). In contrast, validation in untargeted metabolomics assumes a different character, as the analytical objective is not the precise quantification of predefined analytes but rather the comprehensive and reproducible detection of a large number of metabolic features without prior structural knowledge. Consequently, greater emphasis is placed on signal reproducibility, retention time stability, mass spectral quality, control of instrumental drift, and overall consistency of the analytical system, rather than on classical quantitative validation parameters.

**Figure 4:**
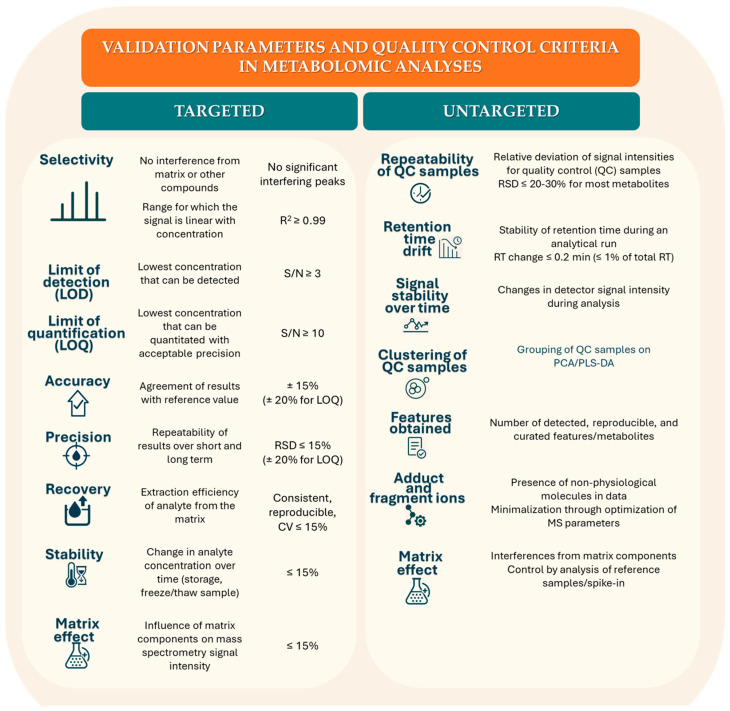
Integrated overview of validation parameters and quality control criteria applied in targeted and untargeted metabolomic analyses. For targeted quantitative assays, acceptance thresholds for target metabolite quantification (LLOQ, accuracy, and precision) were defined according to the FDA Bioanalytical Method Validation Guidance for Industry and the EMA Guideline on Bioanalytical Method Validation.

To provide a coherent overview of these complementary validation concepts, the validation parameters applied in targeted metabolomic analyses and the key quality indicators used for assessing data quality in untargeted metabolomics were integrated into a single schematic (Figure 4). The figure summarises the critical criteria used to verify method performance, signal stability, and reproducibility, thereby providing a unified framework for assessing the analytical robustness of metabolomic studies across different experimental designs. In untargeted metabolomics, quality control (QC) plays a particularly crucial role in ensuring data credibility and reproducibility. Standard practice involves the use of QC samples, most commonly prepared as pooled mixtures of all analysed biological samples, which are injected at regular intervals throughout the analytical sequence [113,114]. This approach enables continuous monitoring of instrument performance, detection of temporal signal drift, and assessment of analytical variability. Quantitative indicators, such as the relative standard deviation (RSD%) of signal intensities calculated for QC samples, are widely used for data quality assessment and should typically remain below 20–30% for the majority of detected features [115]. Multivariate statistical techniques, including principal component analysis (PCA) and partial least–squares discriminant analysis (PLS–DA), further support visual evaluation of QC sample clustering, providing evidence of data homogeneity and the absence of significant batch effects or instrumental instability.

Additional elements essential for ensuring high–quality untargeted metabolomic data include randomisation of sample injection order and balanced sample distribution across analytical batches, which together minimise systematic errors and matrix–related effects [116]. The application of appropriate data processing strategies, such as signal normalisation, drift correction, and filtering of low–quality or irreproducible features, further enhances data robustness and interpretability. Collectively, the implementation of rigorous validation and QC procedures constitutes the foundation for generating high–quality metabolomic datasets suitable for downstream statistical analysis, biomarker discovery, sample classification, and evaluation of therapeutic responses [117].

## 5. Future Perspectives and Research Directions

Analytical platforms based on GC and LC coupled with mass spectrometry (MS) enable the selective separation and subsequent quantification of a broad spectrum of neuroactive compounds, including neurotransmitters, energy metabolites, intermediates of the tryptophan–kynurenine pathway, nucleotides, and lipids, as well as antiepileptic drugs and their metabolites. Of particular importance are neuroactive compounds present at very low concentration levels and characterised by closely related structural features.

Despite substantial advances in chromatographic methodologies, the translation of metabolomic findings into routine clinical practice remains limited. Future research should prioritize the standardisation and harmonisation of analytical workflows, quality control strategies, and validation criteria to ensure interlaboratory reproducibility and regulatory acceptance. Furthermore, metabolomic analyses of postmortem brain tissue from patients with epilepsy may represent a valuable complementary approach, enabling detailed mapping of neuroactive steroids and other neuromodulatory metabolites across distinct brain regions implicated in epileptogenesis. Such strategies may provide novel insights into region–specific metabolic dysregulation and its association with seizure susceptibility. Another important research direction involves the integration of multidimensional metabolomic data with advanced computational methodologies, including machine learning and artificial intelligence (AI). The application of AI–driven data mining, multivariate pattern recognition, and network–based analyses may substantially enhance biomarker discovery by facilitating the integration of metabolomic profiles with clinical, neuroimaging, electrophysiological, and genetic data. These integrative approaches are likely to prove highly valuable for prognostic assessment and prediction of therapeutic response in drug–resistant epilepsy [118,119,120].

## 6. Conclusions

Progress in chromatographic techniques in recent decades has significantly broadened research possibilities in the pathophysiology of epilepsy, allowing for increasingly precise analysis of neurotransmitters, energy metabolites, gut–brain axis compounds, inflammatory biomarkers, and metabolites of antiepileptic drugs. Both gas chromatography and liquid chromatography, particularly in combination with mass spectrometry of high sensitivity and selectivity, have become key tools enabling the characterisation of complex metabolic disturbances accompanying epileptogenesis and identification of potential diagnostic and prognostic indicators.

Recent advances in epilepsy biomarker research have identified key metabolites across several categories: neurotransmitters (glutamate, GABA, dopamine, serotonin), energy metabolites (ATP, ADP, AMP, adenosine, and glucose), tryptophan pathway metabolites (tryptophan, kynurenine, kynurenic acid, and quinolinic acid), and other crucial compounds, including choline, cholesterol, and N–acetylaspartate (NAA). These biomarkers have been characterised using advanced analytical techniques including liquid chromatography–tandem mass spectrometry (LC–MS/MS), high–performance liquid chromatography with electrochemical or fluorescence detection (HPLC–ECD and HPLC–FLD), gas chromatography–mass spectrometry (GC–MS/MS), high–resolution mass spectrometry (HRMS), and proton nuclear magnetic resonance spectroscopy (^1^H–NMR). Analysis of these metabolites in brain tissue, plasma, and urine samples reveals disruptions in neurotransmitter balance, energy metabolism, and oxidative stress pathways that are fundamental to epileptogenesis.

The collected data indicate that metabolomics based on chromatographic techniques constitutes a promising approach to the search for novel epilepsy biomarkers, which may in the future support both the diagnostic process and treatment monitoring, as well as contribute to improved patient stratification and, above all, therapy personalisation. Particularly valuable are HRMS, GC–or LC–MS/MS methods, and hybrid analytical platforms, enabling simultaneous analysis of polar and non–polar compounds over a wide concentration range. Non–invasive and semi–invasive techniques, such as volumetric absorptive microsampling (VAMS) and dried blood spots (DBSs), are also gaining increasing importance and may in the future find application in patient self–monitoring and ambulatory treatment monitoring.

Despite significant development, the available data remain inconsistent, and interpretation of many results is impeded by the lack of standardised analytical protocols, different sample preparation methods, heterogeneity of studied populations, and a shortage of validation studies confirming the clinical value of proposed biomarkers. A key challenge also remains the translation of metabolomic research results into clinical practice, encompassing analytical and clinical validation, determination of cut–off values, development of interpretative algorithms, and integration of results with other diagnostic tools.

In summary, chromatographic techniques constitute the foundation of contemporary metabolomic research in epilepsy and have the potential to become an integral element of future diagnostics and personalised therapy. Their further development, particularly towards high–resolution hybrid platforms, methodology directed at specific metabolic pathways, and standardisation of protocols, may significantly contribute to the improvement of prognosis, quality of life, and treatment efficacy in patients with epilepsy.

## Figures and Tables

**Table 4:** Application of analytical methods employing chromatographic techniques in research on the search for markers in animal models (mice and rats).

**GC**									
**No.**	**Type of Sample**	**Analytical Technique**	**Column**		**Temperature Programme**		**Detector**	**Determined Analytes**	**Ref.**
1	Urine	SPME–GC–MS	InertCap Pure–WAX		40 °C (10 min) → ↑ 5 °C/min → 240 °C (10 min)		Q/QQQ	VOCs	[39]
2	Cerebral cortex	GC–MS	HP–5 MS		Not reported		MS EI	β–Hydroxybutyrate, medium-chain fatty acids, metabolites	[85]
3	Hippocampal extracellular fluid–microdialysate	GC–MS	HP–5 MS		65 °C (1.3 min) → ↑ 25 °C/min → 240 °C → ↑ 10 °C/min → 300 °C (3 min)		Q	Glutamate, glutamine	[48]
**LC**									
**No.**	**Type of Sample**	**Analytical Technique**	**Detector**	**Column**	**Column Temp. [°C]**	**Mobile Phase**	**Elution Programme**	**Determined Analytes**	**Ref.**
1	Brain	HPLC	ECD	Kinetex F5	35	0.07 M KH_2_PO_4_, 20 mM citric acid, 5.3 mM OSA, 100 mM EDTA, 3.1 mM TEA, 8 mM KCl, 11% (*v*/*v*) methanol, pH 3.2 ± 0.1, filtered through 0.22 µm cellulose acetate	Isocratic	Dopamine, noradrenaline, serotonin, 3,4–dihydroxyphenylacetic acid, homovanillic acid, 5–hydroxyindoleacetic acid, vanillylmandelic acid, 3–methoxytyramine, 4–hydroxy–3–methoxyphenylglycol	[30]
2	Hippocampus	HPLC	FLD	Ymc–pack ODS–AQ	Not reported	A: 12.5 mM phosphate buffer pH 6.93 with NaOH and tetrahydrofuran (98.9:1.1); B: methanol, ACN, H_2_O (35:15:50)	0–1 min→10% B → 1–15 min→50% B → 15–18 min→56% B → 18–26 min→65% B → 26–44 min→100% B → 44–45 min→0% B	Glutamine	[86]
3	Plasma, hippocampus	HPLC	TOF–MS	Phenomenex Kinetex HILIC	Not reported	A: 50% ACN with 5 mM acetic acid; B: 90% ACN with 5 mM acetic acid; pH 5.8	0–2 min (100% B) → 2–2.1 min (100–90% B) 2.1–8.6 min (90–50% B) → 8.6–8.7 min (50–0% B) → 8.7–14.7 min (0% B) → 14.7–14.8 min (0–100% B) → 14.8–24.5 min (100% B)	Nucleosides, nucleotides, purine and pyrimidine metabolites, organic acids and their derivatives, peptides and amino acid–related metabolites	[34]
4	Plasma, hippocampus	HPLC	Q–TOF–MS	Agilent Rapid Resolution SB–C18	60	A: H_2_O with 0.1% formic acid; B: (60:36:4) isopropanol:ACN:H_2_O:0.1% formic acid	0–1 min (30% B) → 1–7 min (30–100% B) → 7–12 min (100% B) → 12–13 min (100–30% B) → 13–17 min (30% B)	Ceramides, glucosylceramides, diacylglycerols, phosphatidylcholines, triacylglycerols and vitamin D metabolites and its derivatives	[34]
5	Hippocampus	HPLC	FLD	EUROSIL bioselect 300A	Not reported	A: 0.1 M phosphate buffer (pH 6.0); B: phosphate buffer + methanol + ACN (40:30:30, pH 6.5)	Not reported	Glutamate, asparagine	[87]
6	Hippocampus, frontal cortex, plasma	LC	Q–TOF–MS	ACQUITY UPLC BEH Amide	Not reported	A: 0.1% formic acid + 10 mM ammonium acetate in 20% can; B: 0.1% formic acid + 10 mM ammonium acetate in 95% ACN	1% A/99% B → 50% A/50% B (6.5 min) → 70% A/30% B (7.5 min) → 1% A/99% B (9.0 min)	Untargeted analysis → homostachydrine	[20]
7	Brain	HPLC	ECD	Zorbax SB–C18	35	14.2 g sodium dihydrogen phosphate + 48 mg EDTA in 735 mL H_2_O → made up to 1000 mL with 235 mL methanol + 30 mL ACN, pH 7.63 (adjusted with perchloric acid)	Isocratic	Glutamate, GABA	[21]
8	Brain	HPLC	ECD	Zorbax SB–C18	35	2.5 g citric acid + 1.7 g sodium phosphate + 5 mg EDTA + 600 µL THF in 450 mL H_2_O→ 225 mg heptanoic acid dissolved in 30 mL methanol, pH 3.8	Isocratic	Dopamine, serotonin	[21]
9	Hippocampus	HPLC	FLD	Chromolith C–18	Not reported	A: 0.1 M citrate–phosphate buffer (pH 6.0); B: Eluent A + 25% methanol	Isocratic	ATP, ADP, AMP, adenosine	[37]
10	Plasma	LC–MS/MS	Qtrap	Phenomenex Luna PFP	30	A: 0.1% formic acid in H_2_O B: 100% ACN	0.0 min (98% A–2% B) → 1.3 min (98% A–2% B) → 7.0 min (40% A–60% B) → 8.0 min (0% A–100% B) → 10.0 min (0% A–100% B) → 12.0 min (98% A–2% B) → 15.0 min (98% A–2% B)	Aminoadipate; saccharopine; pipecolate; glutamic acid; piperideine–6–carboxyl acid; pyridoxal–5–phosphate	[38]
11	Hippocampus	UPLC–HRMS	Q–TOF–MS	Acquity UPLC BEH HILIC	40	A: ACN/H_2_O (60;40, *v*/*v*) with 5 mM ammonium formate and 0.1% formic acid; B: 2–propanol/ACN (90;10, *v*/*v*) with 5 mM ammonium formate and 0.1% formic acid	99% A (0–2 min) → 45% A (8 min) → 1% A (9 min) → 1% A (9.1–13 min) → 99% A (13.1 min) → 99% A (17–25 min)	Polar metabolites: glutamine, N–acetyl–L–aspartate, tryptophan, adenosine, glucose–6–phosphate, Fructose–6–phosphate, fructose–1,6–bisphosphate, dihydroxyacetone phosphate, 2–phosphoglycerate, 3–phosphoglycerate, phosphoenolpyruvate, pyruvate, citrate, 2–oxoglutarate, succinate, fumarate, malate	[42]
12	Hippocampus	UPLC–HRMS	Q–TOF–MS	Acquity UPLC CSH C18	40	A: ACN/H_2_O (95;5, *v*/*v*, 10 mM ammonium formate, 0.1% formic acid); B: ACN/H_2_O (50;50, *v*/*v*, 10 mM ammonium formate, 0.1% formic acid)	60% A (0–2 min) → 57% A (2 min) → 50% A (2.1 min) → 46% A (12 min) → 30% A (12.1 min) → 1% A (18 min) → 60% A (18.1 min) → 60% A (18.1–22 min)	Non–polar metabolites: cholesterol, triglycerides, phosphatidylcholine, phosphatidylserine, tree fatty acids, cholic acid, prostaglandins, leukotrienes, anandamide, 2–arachidonoylglycerol	[42]
13	Extracellular fluid from microdialysis of somatosensory cortex	UHPLC	FLD	Zorbax Eclipse Plus–C18	30	A: methanol–ACN–40 mM phosphate buffer (pH 6.7) (20:2:78; *v*/*v*); B: methanol–ACN–40 mM phosphate buffer (pH 6.7) (50:10:40; *v*/*v*)	0–4 min (0% B) → 4–15 min (0% B → 100% B) → 15–20 min (100% B) → 20–25 min (100% B → 0% B) → 25–28 min (0% B, column equilibration)	GABA, glutamate	[43]
14	Striatum, substantia nigra	UPLC	FLD	BEH Glycan	40	A: 50 mM formic acid, pH 4.4; B: ACN	30% A → 47% A (23 min) → 70% A (30 min) → 30% A (return)	N–glycans	[47]
15	Liver	RP–IP–LC	QQQ 5500 + QTrap	Waters Atlantis T3 C18	Not reported	A: H_2_O/methanol (95:5) + 4 mM DBAA; B: H_2_O/ACN (25:75)	0 min (0% B) → 8 min (80% B) → 13 min (100% B) → 16 min (100% B) → 16.1–21 min (0% B)	Adenine nucleotides, short–chain acyl–CoAs	[88]
16	Liver	LC–MS/MS	QQQ 5500 + QTrap	Waters Xbridge C18	Not reported	A: H_2_O/methanol (95:5) + 4 mM DBAA; B: H_2_O/ACN (25:75)	1 min (0% B) → 8 min (80% B) → 13 min (100% B) → 16 min (100% B) → 16.1–21 min (0% B)	Amino acids	[88]

ACN—acetonitrile; ADP—adenosine diphosphate; AMP—adenosine monophosphate; ATP—adenosine triphosphate; DBAA—dibromoacetic acid; EDTA—ethylenediaminetetraacetic acid; ECD—electron capture detector; FLD—fluorescence detector; H_2_O—water; KCl—potassium chloride; KH_2_PO_4_—potassium dihydrogen phosphate; MS EI—mass spectrometry with electron ionisation; NaOH—sodium hydroxide; OSA—octane sulfonic acid; Q—single quadrupole detector; Q–TOF–MS—quadrupole time–of–flight mass spectrometry; Qtrap—quadrupole linear ion trap mass spectrometer; RP–IP–LC—reversed–phase ion–pair liquid chromatography; short–chain acyl–CoAs—short–chain acyl coenzyme A thioesters; TEA—triethylamine; TOF–MS—mass spectrometry with time–of–flight analyser; UHPLC—ultra–high–performance liquid chromatography; UPLC–HRMS—ultra–performance liquid chromatography–high–resolution mass spectrometry; SPME–GC–MS—solid–phase microextraction gas chromatography mass spectrometry; Q—single quadrupole detector; QQQ—triple quadrupole detector; MS EI—mass spectrometry with electron ionisation.

**Table 5:** Application of analytical methods employing chromatographic techniques in monitoring the levels of drugs and their metabolites in patients with epilepsy in physiological fluids and human tissues.

**GC**									
**No.**	**Type of Sample**	**Analytical Technique**	**Column**	**Temperature Programme**		**Detector**	**Determined Analytes**		**Ref.**
1	DBS	HS–GC–MS	HP–5MS	70 °C (1 min) → ↑ 200 °C (20 °C/min) → ↑ 260 °C (50 °C/min, 5 min)		Q	VPA		[59]
2	DBS	GC–MS	DB5–MS	90 °C (0.2 min) → ↑ 10 °C/min → 120 °C (0.5 min) → ↑ 65 °C/min → 285 °C (0.5 min) → ↑ 10 °C/min → 291 °C (0.2 min) → ↑ 60 °C/min → 300 °C (5 min)		Q	CBZ, VPA, phenytoin		[50]
3	Plasma	AALLME–GC	HP–5	70 °C (2 min) → ↑ 15 °C/min → 200 °C → ↑ 20 °C/min → 300 °C		FID	VPA, 3–heptanone		[18]
4	Serum	BioSPME–GC-MS	Rtx–WAX	80 °C (2 min) → ↑ 25 °C/min → 240 °C (1 min)		Q	VPA		[19]
**LC**									
**No.**	**Type of Sample**	**Analytical Technique**	**Detector**	**Column**	**Column Temp.**	**Mobile phase**	**Elution Programme**	**Determined Analytes**	**Ref.**
1	Plasma, serum	LC–MS/MS	QQQ	Agilent Zorbax Eclipse XDB–C8	40	H_2_O:methanol 90:10 (*v*/*v*) with 0.1% acetic acid (A) and methanol: H_2_O 95:5 (*v*/*v*)	0–1.0 min: 20% B → 1.0–5.0 min: 20%–90% B → 5.0–7.0 min: 90% B → 7.0–8.5 min: 90%–20% B → 8.5–10.0 min: re–equilibration	Phenobarbital	[54]
2	Plasma	HPLC	UV	Phenyl–Hexyl	40	Methanol and KH_2_PO_4_	0 min–75% KH_2_PO_4_ (25 mM, pH 5.1)/25% Methanol, 5 min–70%/30%, 10 min–60%/40%, 15 min–58%/42%, 19 min–30%/70%, 23 min–30% /70%, 23.1 min–75%/25%, 27 min–75%/25%	Phenobarbital, phenytoin, primidone, CBZ, ethosuximide, lamotrigine, oxCBZ, rufinamide, zonisamide, lacosamide, LEV, felbamate and metabolites	[55]
3	Saliva	LC–MS/MS	QQQ	Monolithic	25	A: (0.1% formic acid in H_2_O) and mobile phase; B: (methanol)	Gradient: A:B = 98:2 (*v*/*v*) from 0 to 2 min, A:B = 25:75 (*v*/*v*) from 2.01 to 5 min and A:B = 98:2 (*v*/*v*) from 5.01 to 7 min	Perampanel	[49]
4	Plasma	LC–MS/MS	Qtrap MS/MS	SB–C18	40	A: H_2_O; B: ACN	10% B (1 min) → 50% B (to 3 min) → 50% B (to 4.5 min) → 10% B (hold 2.4 min re–equilibration)	CBZ, lamotrigine, oxCBZ, 10–hdroxyCBZ, LEV, phenytoin, VPA, topiramate, phenobarbital, CBZ–E	[56]
5	Serum	HPLC	UV	C–18	25	Triethylamine in H_2_O (pH 6.5) with ACN (85:15)	Isocratic	LEV	[97]
6	Serum	LC–MS^3^	QQQ in MS^3^ mode	Waters XBridge BEH C18	40	A: 0.1% formic acid in H_2_O; B: methanol	Isocratic (50:50, *v*/*v*)	OxCBZ, 10–monohydroxy derivative	[57]
7	Serum	UPLC	DAD	ACQUITY UPLC BEH C18	30	Phosphate buffer (10 mmol/L, pH 4.0) + methanol (55:45, *v*/*v*)	Isocratic (50:50, *v*/*v*)	Lacosamide, oxcarbazepine, lamotrigine	[58]
8	DBS	LC–MS/MS	MRM	ACQUITY UPLC HSS PFP	Not reported	A: 2 mM ammonium formate + 0.2% formic acid in H_2_O; B: ACN	95% A → 90% A (1.1 min) → 90% A (0.9 min) → 80% A (3 min) → 80% A (0.5 min) → 20% A (4.2 min) → 20% A (0.8 min) → re–equi	Lacosamide, lamotrigine, LEV, brivaracetam, phenytoin, gabapentin, pregabalin, primidone, rufinamide, zonisamide	[51]
9	Plasma, serum	ID–LC–MS/MS	QQQ 6500+ and Q–Trap 6500+	Agilent Zorbax Eclipse XDB–C18	40	A: 2 mM ammonium acetate in Milli–Q H_2_O + 0.1% formic acid; B: 95% methanol + 5% 2 mM ammonium acetate in Milli–Q H_2_O + 0.1% formic acid	Mobile phase flow: 0.6 mL/min for 8 min gradient elution	Primidone	[60]
10	DPS	LC–MS/MS	Qtrap MS/MS	Agilent Poroshell 120 SB–C18	40	A: 0.1% formic acid in H_2_O; B: ACN	0.0–0.5 min (70% A–30% B) → 0.5–2.0 min (30% A–90% B) → 2.0–3.5 min (10% A–90% B) → 3.5–4.0 min (30% A–70% B) → 4.0–6.0 min (70% A–30% B)	VPA	[61]
11	Plasma	HPLC	DAD	LiChroCART^®^ Purospher Star–C18	40	A: 0.1% of 85% orthophosphoric acid in H_2_O (pH 2.79); B: ACN	0–5 min (35% A–65% B) → 5–8 min (35% A–65% B) → 8–9 min (10% A–90% B) → 9–12 min (10% A–90% B)	Perampanel, lamotrigine	[62]
12	Plasma, saliva, hair	LC–MS/MS	MS	Kinetex C18 100A	40	Methanol/H_2_O/formic acid (97:3:0.25, *v*/*v*/*v*)	Isocratic	LEV	[52]
13	Plasma/serum	ID–LC–MS/MS	QQQ 6500+	Agilent Zorbax Eclipse XDB–C8	40	A: 10% methanol in H_2_O + 0.1% acetic acid; B: 95% methanol in H_2_O	0.0–1.0 min (100% A–0% B) → 1.0–3.0 min (65% A–35% B) → 3.0–4.2 min (40% A–60% B) → 4.2–5.2 min (20% A–80% B) → 5.2–8.0 min (0% A–100% B) → 8.0–10.0 min (100% A–0% B)	Topiramate	[63]
14	Saliva	HPLC	DAD	Purospher Star–C18	40	A: Ultrapure H_2_O (Milli–Q); B: ACN	97% A–3% B (0 min) → 93% A–7% B (6 min) → 75% A–25% B (8 min) → 97% A–3% B (13 min)	CBZ, CBZ–E, S–licarbazepine, Lacosamide, LEV	[64]
15	Serum	UHPLC–MS/MS	QQQ	ACQUITY UPLC BEH C18	50	A: Ammonium formate (5 mM, pH 7.5); B: ACN	5% B (0.25 min) → 100% B (5.3 min) → 5% B (5.4 min)	Cannabidiol and its metabolites	[65]
16	Plasma	HPLC	–	C18	25	500 mL H_2_O (pH adjusted to 6.5 ± 0.2 using 85% orthophosphoric acid) 15% ACN	Not reported	LEV	[98]
17	Plasma	LC–MS/MS	QQQ	Acquity UPLC HSS PFP	40	A: 0.1% formic acid in H_2_O; B: 0.1% formic acid in ACN	10% B (start) → 90% B (0.3–1.7 min) → 90% B (1.7–2.6 min) → 10% B (return) → Conditioning (2.5 min)	Cenobamate	[99]
18	Plasma	LC–MS/MS	QQQ	Agilent Poroshell 120 EC–C18	–	A: H_2_O with 0.1% formic acid and 2 mM ammonium acetate; B: ACN with 0.1% formic acid	10% B (start) → 90% B (3.5 min) → 10% B (return) → Conditioning (2 min)	Lacosamide, perampanel, gabapentin, pregabalin, vigabatrin, rufinamide	[100]
19	Plasma, serum	HPLC	UV	Synergi™ Fusion–RP, Phenomenex	20	ACN/methanol/H_2_O (15/15/70, *v*/*v*/*v*)	Not reported	Sulthiame	[101]
20	Plasma	HPLC–MS/MS	MS/MS	Hypersil GOLD C18	50	A: 1 mM ammonium acetate in H_2_O B: Methanol	0–0.5 min (90% A) → 0.5–1.2 min (90% A → 5% A) → 1.2–2.0 min (5% A) → 2.0–2.1 min (5% A → 90% A) → 2.1–4.0 min (system stabilisation)	–	[102]

ACN—acetonitrile; DAD—diode array detector; DPS—dried plasma spot; H_2_O—water; ID–LC–MS/MS—isotope–dilution liquid chromatography–tandem mass spectrometry; KH_2_PO_4_—potassium dihydrogen phosphate; MRM—multiple reaction monitoring; MS/MS—tandem mass spectrometry; Qtrap MS/MS—quadrupole linear ion trap tandem mass spectrometry; QQQ—triple quadrupole detector; UHPLC–MS/MS—ultra–high–performance liquid chromatography–tandem mass spectrometry. AALLME–GC—air–assisted liquid–liquid microextraction gas chromatography; BioSPME–GC–MS—biocompatible solid–phase microextraction–gas chromatography–mass spectrometry; FID—flame ionisation detector; Q—single quadrupole detector.

## Data Availability

No new data were created or analysed in this study.

## Abbreviations

The following abbreviations are used in this manuscript:

^1^H–NMR	proton nuclear magnetic resonance spectroscopy
AALLME	air–assisted liquid–liquid microextraction
ADK	adenosine kinase
ADP	adenosine diphosphate
AED	antiepileptic drug
AI	artificial intelligence
AMP	adenosine monophosphate
AMPA	α–amino–3–hydroxy–5–methyl–4–isoxazolepropionic acid
ATP	adenosine triphosphate
BBB	blood–brain barrier
BioSPME	biocompatible solid–phase microextraction
CBZ	carbamazepine
CBZ–E	carbamazepine–10,11–epoxide
CNS	central nervous system
CSF	cerebrospinal fluid
CV	coefficient of variation
DBSs	dried blood spots
DI/LC–MS/MS	direct injection/liquid chromatography coupled with tandem mass spectrometry
EMA	European Medicines Agency
EVs	extracellular vesicles
FDA	United States Food and Drug Administration
FMN	fluoromethylnitrophenylpropionyl
GABA	γ–aminobutyric acid
GAD	glutamate decarboxylase
GC	gas chromatography
GC–MS	gas chromatography coupled with mass spectrometry
GC–MS/MS	gas chromatography coupled with tandem mass spectrometry
GC–TOFMS	gas chromatography coupled with mass spectrometry with time–of–flight analyser
HCl	hydrochloric acid
HILIC	hydrophilic interaction chromatography
HPLC	high–performance liquid chromatography
HPLC–ECD	high–performance liquid chromatography with electrochemical detection
HPLC–MS/MS	high–performance liquid chromatography coupled with tandem mass spectrometry
HRMS	chromatography coupled with high–resolution mass spectrometry
HS	headspace
HS–GC–MS	headspace gas chromatography coupled with mass spectrometry
ICH	International Council for Harmonisation
KATs	kynurenine aminotransferases
LC	liquid chromatography
LC–MS	liquid chromatography coupled with mass spectrometry
LC–MS/MS	liquid chromatography coupled with tandem mass spectrometry
LC–MS^3^	liquid chromatography coupled with triple mass spectrometry
LD	linear dichroism
LEV	levetiracetam
LLOQ	lower limit of quantification
MAO	monoamine oxidases
MHD	10–monohydroxy derivative
MS	mass spectrometry
MTBSTFA	n–methyl–n– (tert–butyldimethylsilyl)trifluoroacetamide
MTLE–HS	mesial temporal lobe epilepsy with hippocampal sclerosis
NAA	N–acetylaspartate
NMDA	N–methyl–D–aspartate
OPA	ovphthalaldehyde
OXC	oxcarbazepine
PCA	principal component analysis
PLS–DA	partial least–squares discriminant analysis
PNP	paranitrophenyl
QC	quality control
RP–LC	reversed–phase system liquid chromatography
RSD%	coefficient of variation
SE	status epilepticus
SLE	solid–liquid extraction
SPE	solid–phase extraction
SPME	solid–phase microextraction
TMCS	trimethylchlorosilane
UHPLC–MS/MS	ultra–high–performance liquid chromatography coupled with tandem mass spectrometry
UPLC	ultra–performance liquid chromatography
UV	ultraviolet
UV–VIS	ultraviolet–visible
VAMS	volumetric absorptive microsampling
VOCs	volatile organic compounds
VPA	valproic acid
WHO	World Health Organization
βME	β–mercaptoethanol
